# Intraoperative Assessment and Photothermal Ablation of the Tumor Margins Using Gold Nanoparticles

**DOI:** 10.1002/advs.202002788

**Published:** 2021-01-18

**Authors:** Qiaolin Wei, Hamed Arami, Hélder A. Santos, Hongbo Zhang, Yangyang Li, Jian He, Danni Zhong, Daishun Ling, Min Zhou

**Affiliations:** ^1^ The Fourth Affiliated Hospital Zhejiang University School of Medicine Yiwu 322000 P. R. China; ^2^ Institute of Translational Medicine Zhejiang University Hangzhou 310009 P. R. China; ^3^ State Key Laboratory of Modern Optical Instrumentations Zhejiang University Hangzhou 310058 P. R. China; ^4^ Molecular Imaging Program at Stanford Department of Radiology Stanford University Stanford CA 94305‐5427 USA; ^5^ Drug Research Program Division of Pharmaceutical Chemistry and Technology Faculty of Pharmacy University of Helsinki Helsinki FI‐00014 Finland; ^6^ Helsinki Institute of Life Science (HiLIFE) University of Helsinki Helsinki FI‐00014 Finland; ^7^ Pharmaceutical Science Laboratory Åbo Akademi University Turku 20520 Finland; ^8^ Institute of Pharmaceutics College of Pharmaceutical Sciences Zhejiang University Hangzhou Zhejiang 310058 P. R. China; ^9^ Key Laboratory of Cancer Prevention and Intervention National Ministry of Education Zhejiang University Hangzhou 310009 P. R. China

**Keywords:** gold nanoparticles, intraoperative assessment, photothermal ablation, surface‐enhanced Raman scattering imaging, tumor margins

## Abstract

Surgical resection is commonly used for therapeutic management of different solid tumors and is regarded as a primary standard of care procedure, but precise localization of tumor margins is a major intraoperative challenge. Herein, a generalized method by optimizing gold nanoparticles for intraoperative detection and photothermal ablation of tumor margins is introduced. These nanoparticles are detectable by highly sensitive surface‐enhanced Raman scattering imaging. This non‐invasive technique assists in delineating the two surgically challenged tumors in live mice with orthotopic colon or ovarian tumors. Any remaining residual tumors are also ablated by using post‐surgical adjuvant photothermaltherapy (aPTT), which results in microscale heat generation due to interaction of these nanoparticles with near‐infrared laser. Ablation of these post‐operative residual micro‐tumors prolongs the survival of mice significantly and delays tumor recurrence by 15 days. To validate clinical translatability of this method, the pharmacokinetics, biodistribution, Raman contrast, aPTT efficiency, and toxicity of these nanoparticles are also investigated. The nanoparticles have long blood circulation time (≈24 h), high tumor accumulation (4.87 ± 1.73%ID g^−1^) and no toxicity. This high‐resolution and sensitive intraoperative approach is versatile and can be potentially used for targeted ablation of residual tumor after resection within different organs.

## Graphical abstract

The surface‐enhanced Raman scattering nanoparticles provide intraoperative detection and image‐guided cancer surgery. The remaining residual tumors are then ablated by a post‐surgical adjuvant photothermal therapy. Ablation of these residual microtumors, prolongs the survival significantly and reduces tumor recurrence. This high‐resolution and sensitive intraoperative approach is versatile and can be potentially used for targeted ablation of post‐resection residual tumors.

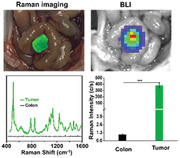

## 1 Introduction

Solid tumors are often treated by surgery and post‐surgical chemo‐ and radiotherapies.^[^ ^1^, ^2^, ^3^ ^]^ However, precise targeting and real‐time intraoperative localization of the tumor margins remains a challenging task, which usually results in tumor recurrence if residual tumor is left within the resected regions. Therefore, it is necessary to resect larger areas of surrounding normal tissues or even whole organ in order to minimize the chances of recurrence. Furthermore, as intraoperative detection of small tumor margin residues is difficult, post‐surgical radiotherapy is often applied to the larger areas, including the normal tissues that surround the resected tumor cavity to prevent recurrence.^[^ ^4^, ^5^ ^]^ Excessive tissue resection and ionizing radiation causes damage (e.g., inflammation, fibrosis, atrophy, and vascular damage), and complications, including loss of organs, mechanical injury, massive hemorrhage, bacterial infection, radiation‐induced heart diseases, and secondary metastatic malignant diseases.^[^ ^6^, ^7^ ^]^ Therefore, a precise surgery of the tumor is crucial, and post‐surgical treatments should also be optimized further to improve the quality of life and reduce the number of deaths of cancer patients with surgically unresectable residual tumors.

Nanoparticles have received much attention in the past decade owing to their unique physical and chemical properties that are suitable for multimodal imaging and therapies, as well as in other biomedical applications.^[^ ^8^, ^9^, ^10^, ^11^, ^12^, ^13^, ^14^ ^]^ Among different nano‐formulations, gold nanoparticles have been widely used in biomedical applications because of suitable biocompatibility, controllable size, and ease of surface modification.^[^ ^15^, ^16^ ^]^ Additionally, gold nanoparticles present surface plasmon that makes them suitable as Raman spectroscopy agents for detecting cancer and imaging with ultrahigh sensitivity (10^−12^–10^−14^ m) and negligible auto‐fluorescence.^[^ ^17^, ^18^, ^19^, ^20^, ^21^, ^22^, ^23^, ^24^ ^]^ Gold nanoparticles of different morphological shapes such as nanorods, nanospheres, and branched nanostructures (e.g., nanostars) exhibit different surface‐enhanced Raman scattering (SERS) characteristics that are specifically tuned for imaging and detection benefits of cancer.^[^ ^19^, ^20^, ^21^, ^22^, ^23^, ^24^ ^]^ In particularly, the branched or urchin shaped gold nanostructures had unique SERS effects that are attributed to the anisotropic distribution of electromagnetic field near their surface.^[^ ^18^, ^25^, ^26^, ^27^, ^28^, ^29^, ^30^, ^31^, ^32^ ^]^ Previous studies have demonstrated the existence of a large electromagnetic field enhancement (so‐called hotspot) at the tips of these branched sites, which subsequently lead to enhanced SERS activity.^[^ ^31^, ^32^ ^]^ In addition, the branched or urchin shaped gold nanoparticles provide larger surface areas (approximately tenfold more compared with smooth nanoparticles) for the conjugation of more Raman tags per nanoparticle, and this results in stronger Raman signals.^[^ ^26^, ^27^ ^]^ Moreover, such plasmonic gold nanoparticles assists in generating controlled heat in response to laser irradiation in different photothermal therapy (PTT) approaches, and these can be potentially used as alternatives or in conjunction with radiation therapy.^[^ ^33^, ^34^ ^]^

Gold nanoparticles have been used for SERS‐based Raman imaging of different types of tumors such as orthotopically implanted brain tumors,^[^ ^31^ ^]^ gastrointestinal lesions,^[^ ^35^ ^]^ endogenous liver tumors,^[^ ^29^ ^]^ and other xenograft tumors.^[^ ^36^ ^]^ Also, multiple studies have reported the use of image‐guided photothermal therapy by gold nanoparticles. However, it has limited imaging resolutions, and sufficient only for measuring volumetric tumors or qualitative monitoring of larger‐size (i.e., 1–2 cm diameter) subcutaneous tumor xenografts by using magnetic resonance imaging, photoacoustic imaging (PAI), or whole‐body fluorescent imaging.^[^ ^28^, ^30^, ^36^ ^]^ Recent advancements in intraoperative Raman microscopy using gold nanoparticles offer high‐resolution Raman image‐guided surgeries that offer more accurate resection of the tumor margin areas.^[^ ^23^, ^31^ ^]^ This accurate tumor resection procedure assists in potentially decreasing the chances of recurrence by removing the proliferating residual tumors, especially when combined with other optically‐tuned therapeutic modalities such as PTT. In a recent study, these two modalities were coupled and demonstrated the effectiveness of Raman image‐guided surgery and intraoperative PTT for detection and ablation of metastatic micro‐tumors in mice.^[^ ^32^, ^37^ ^]^ However, additional studies are required to validate the feasibility, accuracy and safety of this technique for simultaneous detection and ablation of post‐resection residual tumor margins, especially for those tumors that are associated with essential organs and have limited accessibility to surgeons, such as colon. Such a precise combination therapeutic approach potentially decreases the recurrence rate, leading to prolonged survival as post‐resection residual tumors are believed to be the major cause of recurrence. In addition, further systematic investigations are necessary to delineate the in vivo characteristics of SERS nanoparticles (i.e., biodistribution and pharmacokinetics) for delivering sufficient amount of nanoparticles to tumors for improving the accuracy and effectiveness of such intraoperative tumor resection and PTT approaches.

We herein first evaluated gold nanoparticles for intraoperative Raman‐guided resection of subcutaneous and orthotopic ovarian and colon tumors until no more SERS signals could be detected within the tumor bed. Next, a post‐surgical PTT was then used for selective ablation of tumor margin residues in mice with ovarian cancer. The tumor growth and recurrence after therapy were monitored to investigate the efficacy and feasibility of the photothermal ablation technique. In addition, the in vivo pharmacokinetics, biodistribution, and toxicity properties of these nanoparticles were further evaluated by PAI and positron emission tomography (PET). These results showed the versatility of this strategy in eliminating the tumor margin residues more effectively and prolonging the survival of mice significantly by postponing the recurrence to at least 15 post‐operative days. This approach provides insights for better management of post‐resection recurrence associated with different types of malignant tumors.

## 2 Results and Discussion

### 2.1 Characterization of the Nanoparticles

Hydrogen peroxide (H_2_O_2_) was sued as an efficient reducing agent and sodium citrate as stabilizing agent for synthesizing urchin‐like gold nanoparticles (Au‐Ur NPs). transmission electron microscopy (TEM) results showed that these nanoparticles had a nanostructure that was similar to that of sea urchins (Figure S1A–C, Supporting Information). Adjustment of sodium hydroxide concentration during the synthesis allowed for tuning of the mean particle diameters to ≈55, ≈72, and ≈92 nm. In addition, the Au‐Ur nanoparticles showed good dispersion in aqueous solutions with distinguishable colors due to surface plasmon resonance characteristics of Au‐Ur nanoparticles. The UV–vis absorbance spectra (Figure S1D–F, Supporting Information) showed that the maximum absorption wavelength of the nanoparticles depends on their size, wherein the larger size nanoparticles have greater red‐shifts. ≈72 nm nanoparticles that showed a sharp absorption peak at 808 nm were aligned well with the wavelength of the near‐infrared (NIR) laser system used for PTT and then used for all the subsequent experiments reported in this study. The branched urchin‐like nanostructure and lattice fringes that represent the crystalline phase of these nanoparticles are shown in Figure S1G,H, Supporting Information. The distance between the adjacent lattice fringes was 0.236 nm, and this was well consistent with the {111} interplanar spacing of cubic Au lattice structure. The urchin‐like nanostructure of these nanoparticles was further confirmed by scanning electron microscopy (SEM), which demonstrated a uniform spherical shape with rough surface with an average diameter of ≈72 nm (Figure S1I, Supporting Information).

**Figure 1:**
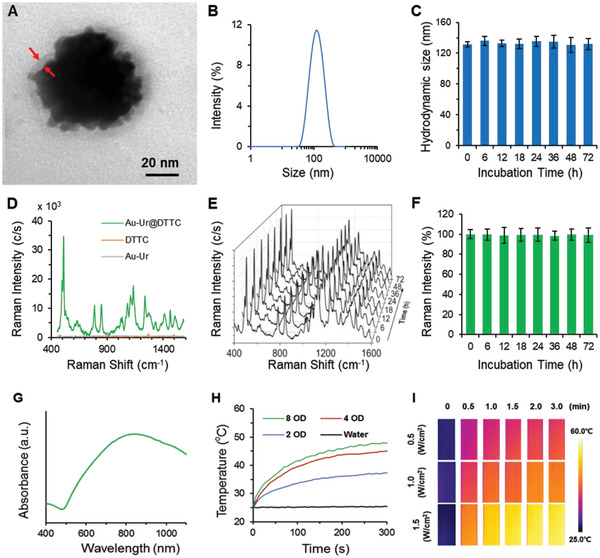
Characterization of Au‐Ur@DTTC nanoparticles. A) Typical TEM image of a Au‐Ur@DTTC nanoparticle. The arrows denote a semi‐transparent 4–6 nm thick coating. B) Dynamic light scattering analysis. C) Size stability in serum over a period of 72 h (*n* = 3). D) Raman spectra of Au‐Ur, DTTC, and Au‐Ur@DTTC nanoparticles. E,F) Stability of the Raman signals and intensity of the 507 cm^−1^ peak after incubation of the nanoparticles in serum over a period of 72 h (*n* = 3, 785 nm laser, 150 mW, 5× objective, 0.5 s integration time). G) UV–vis absorbance spectrum. H) Increase in temperature due to irradiation of different concentrations of Au‐Ur@DTTC nanoparticles with the 808 nm laser. I) Thermal images of Au‐Ur@DTTC nanoparticles (concentration ≈4 OD) under irradiations with different laser powers.

The effects of nanoparticles on SERS has been extensively investigated by different biomedical applications, mainly based on highly‐sensitive detection of the nanoparticles (i.e., up to 10^−12^ m) with multiplexing capabilities.^[^ ^38^ ^]^ Diethylthiatricarbocyanine iodide (DTTC) is a common infrared dye molecule that has been used as a reporter adsorbed on the surface of the gold nanoparticles in SERS applications.^[^ ^39^, ^40^ ^]^ The DTTC molecules were conjugated to the surface of the urchin‐like nanoparticles by using an Au‐S bonding to create an active SERS probe (Au‐Ur@DTTC), wherein the DTTC signal can be used for specific detection of nanoparticles by SERS imaging technique. TEM analysis showed a thin shell with a thickness of about 4–6 nm for covering the surface of Au‐Ur nanoparticles and the energy‐dispersive X‐ray spectroscopy (EDS) mapping images indicated that the coating layer was a mixture of DTTC and PVP molecules (**Figure** **1**A and Figure S2, Supporting Information). Dynamic light scattering (DLS) showed that the hydrodynamic diameter of the Au‐Ur@DTTC nanoparticles was around 131 nm (Figure 1B), and was quite stable in serum solution (Figure 1C), with negligible changes in size after 72 h of incubation (*p*> 0.05 relative to time 0 h). The zeta‐potential of the Au‐Ur@DTTC nanoparticles was changed from −29.5 ± 4.1 to −8.5 ± 1.3 mV due to the presence of DTTC molecules on the surface of Au‐Ur nanoparticles (Figure S3A,B, Supporting Information). EDS spectrum (Figure S3C, Supporting Information) showed the co‐existence of Au, S, N, and O elements in the Au‐Ur@DTTC nanoparticle complexes. The presence of S, O, and I elements, which attributed to DTTC molecules, were observed in X‐ray photoelectron spectroscopy (XPS) spectra (Figure S3D, Supporting Information), further confirming the effective conjugation of DTTC molecules to Au‐Ur nanoparticles. Meanwhile, the X‐ray diffraction (XRD) pattern of the Au‐Ur@DTTC nanoparticles (Figure S3E, Supporting Information) verified the presence of cubic Au phase (JCPDS No. 04‐0784) and the absorbed DTTC molecules did not compromise the crystallinity of gold nanoparticles.

Raman spectra of Au‐Ur nanoparticles, DTTC and Au‐Ur@DTTC nanoparticles are shown in Figures 1D, in which the DTTC powder showed a very weak signal, and Au‐Ur@DTTC nanoparticles exhibited 10^4^‐fold enhanced Raman signals, even though a significantly higher concentration of DTTC powder was used when compared with Au‐Ur@DTTC nanoparticles. The Raman peaks were observed at 507, 786, 848, and 1109 cm^−1^, which are assignable to different vibration modes of DTTC molecules (Figure S3F, Supporting Information), and this was obviously different as the result of SERS effects on the surface of specific urchin‐like nanostructure of the nanoparticles. In this study, the characteristic vibrational modes at 507 cm^−1^ for Raman imaging were chosen owing to its strong signal and sharp peak (half‐peak width = 5 nm). The Raman signal of Au‐Ur@DTTC nanoparticles was consistent during incubation in the serum for 72 h (i.e., biological stability, Figure 1E,F) and continuous irradiation by 785 nm laser (150 mW) for up to 30 min (i.e., photostability, Figure S4, Supporting Information). Furthermore, the Au‐Ur@DTTC nanoparticles showed a strong absorption in the near infrared region (Figure 1G, peak at 825 nm), indicating potential PTT capabilities under 808 nm laser irradiation (Figure 1H,I).

### 2.2 In Vivo PAI, PET Imaging, Pharmacokinetics, and Biodistribution

PAI has been developed based on the acoustic effects of light‐absorbers and offers remarkable increase in imaging depth and spatial resolution.^[^ ^41^, ^42^ ^]^ The Au‐Ur@DTTC nanoparticles have a high absorbance in the NIR region (Figure 1G), making it as a strong contrast agent for photoacoustic (PA) imaging. Representative PA images and corresponding PA intensities were present in Figure S5A,B, Supporting Information, which demonstrated that the Au‐Ur@DTTC nanoparticles were initially accumulated in the tumor tissues, allowing tumor visualization at 1 h, which then continued to increase after 5 and 24 h due to enhanced permeability and retention (EPR) effect. Also, the Au‐Ur@DTTC nanoparticles were radiolabeled with ^64^Cu ([^64^Cu]Au‐Ur@DTTC nanoparticles, Figure S6, Supporting Information) and PET imaging was performed to determine the pharmacokinetics and biodistribution of these nanoparticles. This method has been extensively used to quantify the in vivo distribution of the nanotherapeutic particles due to its high tracer mass sensitivity.^[^ ^43^ ^]^ PET images obtained at various time points after intravenous administration of [^64^Cu]Au‐Ur@DTTC nanoparticles are presented in Figure S5C, Supporting Information, and the radioactivity at SKOV3 tumor site (Figure S5D, Supporting Information) was shown to gradually increase at 24 h post‐injection. The pharmacokinetic profile of the nanoparticles at different time points after injection was shown in Figure S5E, Supporting Information, and the blood clearance pharmacokinetic profile of [^64^Cu] Au‐Ur@DTTC nanoparticles followed a two‐compartment mathematical model,^[^ ^44^, ^45^ ^]^ with the first and second phase blood circulation half‐lives at 0.66 ± 0.21 and 14.22 ± 0.25 h, respectively. The quantitative ex vivo biodistribution measurements (Figure S5F, Supporting Information) demonstrated that the intensity of PET signals in tumor site was 4.87 ± 1.73% ID g^−1^, and this was in agreement with the in vivo PET results (Figure S5C, Supporting Information).

### 2.3 In Vivo Raman Imaging in Subcutaneous SKOV3 Ovarian and CT26 Colon Tumor Models

**Figure 2:**
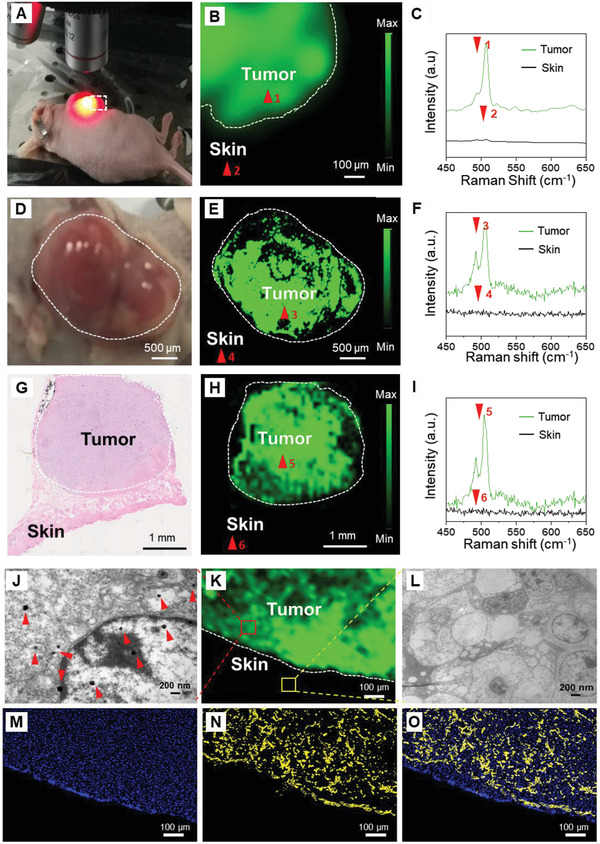
Raman imaging of representative SKOV3 and CT26 tumor margins 24 h post‐injection of the Au‐Ur@DTTC nanoparticles. A) Photograph of a mouse bearing subcutaneous SKOV3 tumor and B) Raman image of this tumor. C) The corresponding Raman spectra within the tumor (red arrow 1), and adjacent skin tissue (red arrow 2). D) Photograph of a mouse with subcutaneous CT26 tumor and E) Raman image of this tumor. F) The corresponding Raman spectra within the tumor (red arrow 3), and adjacent skin tissue (red arrow 4). G) Histology image of a H&E stained CT26 tumor section, showing the tumor core and its surrounding skin tissue. H) The corresponding Raman image of the tumor (red arrow 5) and adjacent skin (red arrow 6). The white dashed lines in (B–E) and (H) denote the tumor margins. J,K) TEM and Raman microscopy images of the CT26 tumor (red arrows in the TEM image are the Au‐Ur@DTTC nanoparticles). L) TEM image of the skin tissue, surrounding the tumor shown in (K), red and yellow boxes denote the tumor and normal tissue. The boundary (white dotted line) between the tumor and adjacent normal tissue was outlined by Raman imaging. A large number of Au‐Ur@DTTC nanoparticles were confined to the tumor site and almost no nanoparticles were found in adjacent skin tissue. M) Fluorescent (the tissues were stained by DAPI), N) dark field, and O) merged Fluorescent‐Raman images of the tumor tissue. The corresponding dark‐filed image also demonstrates high uptake of the nanoparticles by the tumor. Raman images were captured under 785 nm laser (150 mW, 5× objective, 0.2 s integration time, and the total time is 5–25 min).

The in vivo Raman imaging efficiency of our Au‐Ur@DTTC nanoparticles was evaluated using mice without any tumors. All measurements were performed by subcutaneous injections of phosphate buffered saline (PBS), Au‐Ur, and Au‐Ur@DTTC nanoparticles (*n* = 3) into live mice (Figure S7, Supporting Information), and the results showed that the in vivo Raman spectrum of the position injected with Au‐Ur@DTTC nanoparticles demonstrated a clear SERS signature when compared with the skin tissue injected with PBS and Au‐Ur. These results suggest that Au‐Ur@DTTC nanoparticles are suitable as high‐contrast agent in cancer imaging by Raman spectroscopy. The biodistribution studies (Figure S5F, Supporting Information) suggested that Au‐Ur@DTTC nanoparticles had the highest uptake (4.87 ± 1.73% ID g^−1^) by the tumors 24 h post‐injection. Therefore, Raman imaging of the subcutaneous SKOV3 tumors (*n* = 5) was performed at this post‐injection time point, and Raman imaging was captured by the StreamLine ultra‐fast imaging mode of our Raman system and Raman images were generated using the peak at 507 cm^−1^ (**Figure** **2**A,B, Supporting Information). No Raman signal was detected at the skin tissues near the tumor, which was further confirmed by the representative skin Raman spectrum shown in Figure 2C. The ratio of Raman signals at the tumor were significantly higher than those signals at the adjacent skin tissues (tumor = 39 500 versus skin = 150, around 263‐fold enhancement) (Figure S8, Supporting Information).

Also, the in vivo Raman imaging performance of Au‐Ur@DTTC nanoparticles was tested in mice with intact CT26 colon tumors (*n* = 3, Figure 2D–F), which enabled us to delineate the tumor from normal healthy tissues. The images of hematoxylin‐eosin (H&E) stained CT26 tumor section and its adjacent skin tissue were shown in Figure 2G, the Raman image was shown in Figure 2H and corresponding Raman spectra in Figure 2I, which demonstrated a significantly stronger Raman signal within the whole tumor tissue when compared with no visible signals at the nearby skin area, which further verified the feasibility of Raman imaging for accurate tumor detection.

Finally, the tumor margins (i.e., the interface of the tumors and their surrounding normal tissues) were assessed in the tissues obtained from the mice discussed above. As shown in Figure 2J–O, the boundary (white dotted line in Figure 2K) between the tumor and normal tissue was clearly outlined by Raman mapping, demonstrating the efficiency and selective retention of the nanoparticles into the tumor. Additionally, TEM images of these tissues have confirmed the presence of a large number of Au‐Ur@DTTC nanoparticles within the tumors and with no nanoparticles observed in the adjacent normal tissue (Figure 2J,L). The corresponding dark‐filed images demonstrated a similar conclusion with that of Raman imaging (Figure 2M–O). Overall, these results indicate that our Raman imaging approach can be used for imaging and image‐guided resection of the tumors, relying on the Raman signals and selective distribution of Au‐Ur@DTTC nanoparticles in the tumor tissues, which will be discussed in a more detailed manner in the following sections.

### 2.4 Precise Image‐Guided Resection of the Subcutaneous SKOV3 Ovarian and CT26 Colon Tumors

**Figure 3:**
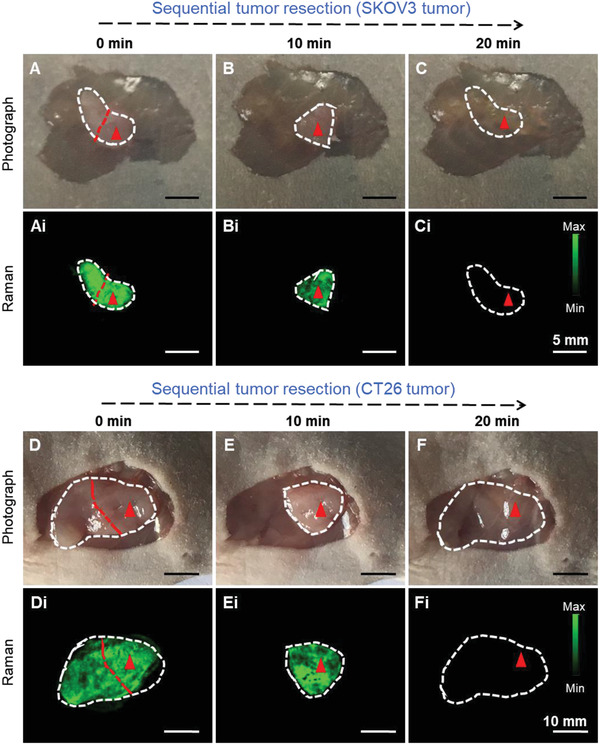
Raman image‐guided intraoperative resection of subcutaneous SKOV3 ovarian and CT26 colon tumors. A–C) Photographs of the subcutaneous SKOV3 tumor after removing the skin and Ai–Ci) corresponding Raman images from the same areas shown in (A–C) at different stages of the resection. Raman signals were only observed within the tumor, but not in surrounding skin or muscle tissues. This enabled us to do a precise tumor resection surgery using Raman imaging. D–F) Photographs of the subcutaneous CT26 tumor and Di–Fi) counterpart Raman images. Similar to (Ai–Ci), Raman signals were only observed within the tumor, which enabled sequential resection of the tumors. The tumors are highlighted by white dotted lines, and Raman images were captured under 785 nm laser (150 mW, 5× objective, 0.2 s integration time, and the total time is 8–20 min).

Uniform and selective accumulation of Au‐Ur@DTTC nanoparticles within the tumors assisted in delineating the tumor margins by Raman imaging. Raman image‐guided tumor resection was performed in mice models with subcutaneous SKOV3 ovarian or CT26 colon tumors (0.5–500 mm^3^) under 785 nm laser, which not only minimizes auto‐fluorescence and photo‐toxicity, but also helps to detect tumor foci (0.6 mm) that was hidden below (3 mm) the normal tissue at 24 h after intravenous administration of Au‐Ur@DTTC nanoparticles. First, the whole intact tumor was scanned using Raman imaging, and with each sequential resection step a high congruency was observed between the residual tumor tissues (**Figure** **3**A–C and 3D–F for SKOV3 and CT26 tumors, respectively) and the presence of Raman signals (Figure 3Ai–Ci and 3Di–Fi). Furthermore, after complete resection of the tumor (Figure 3C,F), no Raman signals were detected (Figure 3Ci,Fi) in the resection bed (*n* = 3). Additionally, the penetration depth of the Raman signal was evaluated by covering the nanoparticles with different thicknesses of chicken tissues and then performing the Raman spectroscopy (Figure S9, Supporting Information). The results showed that the Raman signals penetrated for about 4 mm in the chicken tissues and the intensity of Raman signals was lowered and lowered with increasing thickness as shown in Figure S9B,C, Supporting Information. This certified that the Raman imaging technique under 785 nm laser has advantages for detecting signals under the skin and avoiding skin interference.

### 2.5 In Vivo Raman Imaging of Orthotopic CT26 Colon Tumors

**Figure 4:**
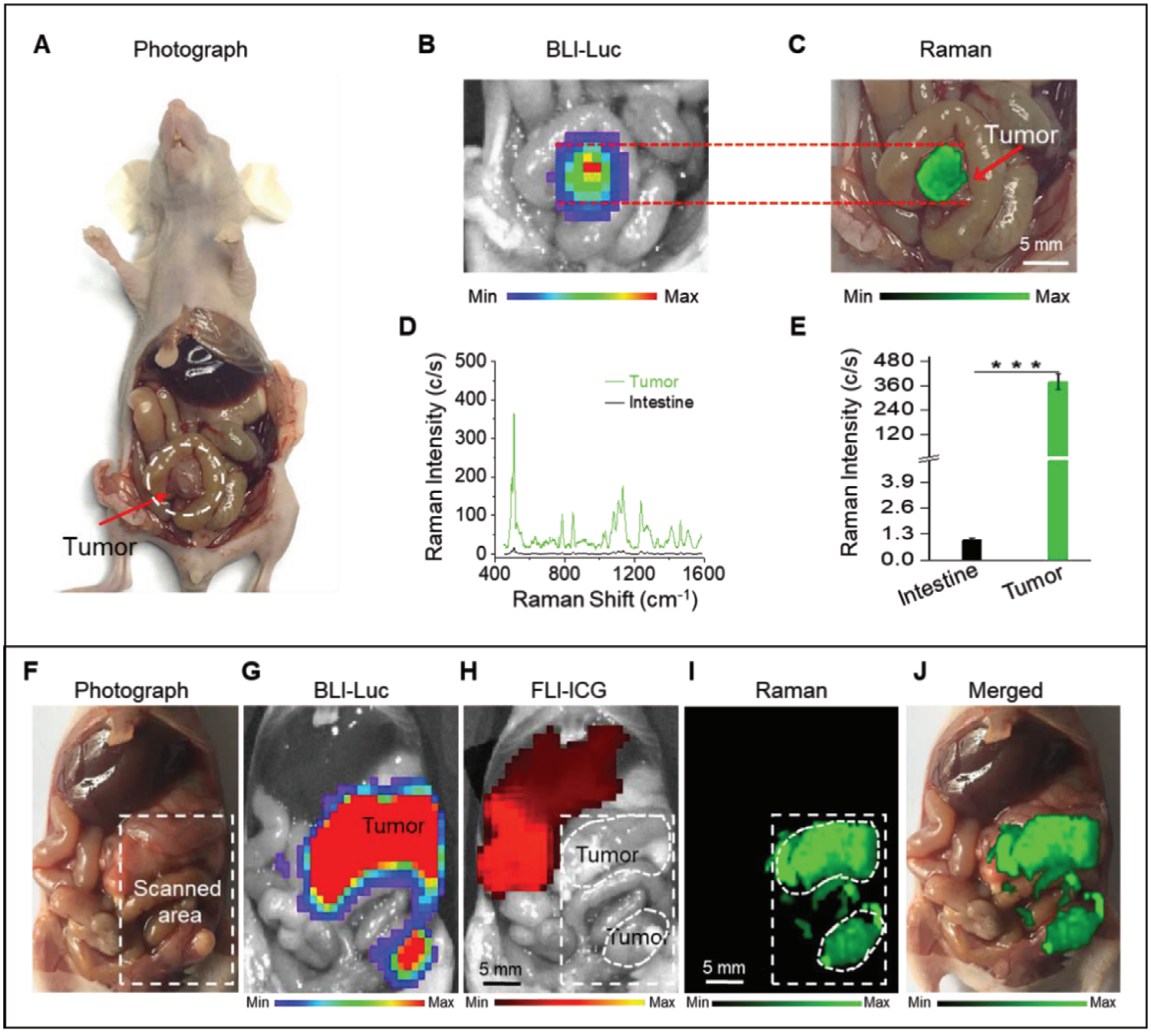
A–G) Raman imaging of orthotopic CT26 colon and F–J) SKOV3 ovarian tumors. A,B) Photograph and in situ BLI of a mouse with orthotopic CT26 colon tumor and C) Raman image of the tumor. D) Raman spectra and E) Raman signals of the tumor and surrounding intestine tissues, showing significant accumulation of the nanoparticles within the tumor, without any detectable uptake in the normal intestine tissues. F) Photograph of the exposed abdomen shows multiple SKOV3 tumors, which were confirmed by in situ BLI of the tumor shown in (G). Raman images in (I) match well with the tumor BLI signal (G), showing retention of the nanoparticles within the tumors and helped delineation of the tumor margins. However, H) the ICG fluorescent signals were only observed at liver, indicating that all ICG were cleared from the body and accumulated in liver. Fluorescence image in (H) was captured 24 h after intravenous injection of ICG. J) The overlaid version of the Raman image on its counterpart abdominal photograph. The selected region for Raman imaging is shown with a white‐color dashed rectangle. Raman images were captured under 785 nm laser (150 mW, 5× objective, 0.2 s integration time, and the total time is ≈10 min for (C) and ≈32 min for (I), respectively). Data are presented as the mean ± SD, *p* values were calculated by Student's *t*‐test, (*n* = 5, ***p < 0.001, relative to the control group).

In situ Raman imaging was used for exposing the abdominal cavity to delineate the orthotopic CT26 (Luc^+^) colon tumors in mice (*n* = 3, **Figure** **4**A). The Raman signals were matched well with that of the bioluminescence imaging (BLI) results regarding the exact location of the tumor lesions (Figure 4B,C). Similar to our previous results, the Raman spectra at the tumor site was intense, showing almost no detectable signals at the surrounding normal tissues (Figure 4D). The signal intensity of 507 cm^−1^ band was 380‐folds higher at the tumor area when compared with surrounding non‐cancerous intestinal sections (Figure 4E).

### 2.6 In Vivo Raman Imaging and Comparison with Indocyanine Green Fluorescence Imaging in Orthotopic SKOV3 Ovarian Tumor Model

Indocyanine green (ICG) is a Food and Drug Administration approved contrast agent that is widely used for monitoring angiography procedures and fluorescence image‐guided surgeries.^[^ ^46^ ^]^ An orthotopic SKOV3 (Luc^+^) tumor‐bearing mouse model (*n* = 3) was used to assess whether Raman imaging of the tumors with Au‐Ur@DTTC nanoparticles has any advantages when compared with tumor fluorescent imaging (FLI) by using ICG contrast. ICG and Au‐Ur@DTTC nanoparticles were co‐injected via tail vein 24 h before imaging. The animals were euthanized, and the abdomen was surgically exposed for Raman and fluorescence imaging. A representative mouse was shown in Figure 4F–J. The results of disseminated SKOV3 tumors were presented in the mice abdomen (Figure 4F) and the corresponding BLI (Figure 4G) results were shown. The FLI indicated that the ICG signals were only detected in the liver (Figure 4H), while Raman mapping (Figure 4I,J) assisted in distinguishing the normal liver parenchyma from the tumors. Furthermore, Raman signals have enabled a precise outlining of the tumor lesions that were not detectable by visual inspection or palpation, and no ICG signals could be seen within the tumor. This was mainly due to the EPR effect of the nanoparticles when compared with small ICG molecules that tend to excrete from the tumor after uptake.^[^ ^47^ ^]^ The tumor lesions and surrounding normal tissues were further identified by histopathologic analyses (Figure S10, Supporting Information).

### 2.7 Image‐Guided Intravital Surgery of the Orthotopic Tumors

**Figure 5:**
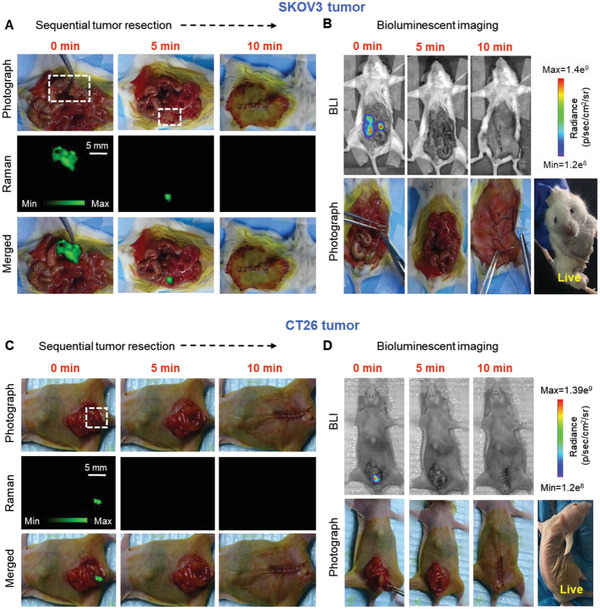
Raman image‐guided resection of the SKOV3 ovarian and CT26 colon tumors. A) Abdominal photographs and their corresponding Raman images during our step‐by‐step Raman‐guided resections surgery, and B) BLI images of the same mouse before and after resection of orthotopic SKOV3 tumors using Raman images shown in (A). Mice photographs and their corresponding C) Raman and D) BLI images at different stages of the Raman image‐guided resection of orthotopic CT26 tumor. Raman images were captured under 785 nm laser (150 mW, 5× objective, 0.2 s integration time, and 5–15 min in total).

Based on the results that Raman imaging assists in detecting the tumor accurately, the capability of Raman imaging approach was evaluated in intraoperative resection of orthotopic tumors models: SKOV3 ovarian (*n* = 3) tumor model and CT26 colon (*n* = 3) tumor model. The whole SKOV3 tumor was first visualized using Raman imaging (**Figure** **5**A), and noted a high congruency between the amount of residual tumor tissues and the presence of Raman signals, within the detection range of our Raman system. The tumor resection was initiated based on our preliminary visual inspections, and then the Raman imaging enabled us to find additional micro‐tumor foci (Figure 5A) in the resection bed, which act as indicators of the residual tumor margins. These residual tumor foci were resected based on continuous Raman imaging until surgical resection cannot be performed. As shown in Figure 5B, no bioluminescence signal was found after surgery and suturing, and the mice were alive till day 7 post‐surgery. Also, a similar Raman image‐guided surgery was carried out on orthotopic CT26 colon tumor model (Figure 5C) and obtained similar results. No bioluminescence signal was observed after the surgeries (Figure 5D). Taken together, Raman and bioluminescent images (representing the nanoparticles and Luc^+^ tumor cells) matched well at different stages of tumor resection, indicating that Au‐Ur@DTTC nanoparticles have been selectively accumulated within the tumors. Moreover, the nanoparticles enabled a precise detection of microscopic tumor residues (<1 mm in diameter) in view of their enhanced Raman signals.

### 2.8 Adjuvant PTT in both Subcutaneous and Orthotopic SKOV3 Ovarian Tumor Model

The residual micro‐scale tumors lead to recurrence and metastasis and decrease of the survival rate significantly. Cancer patients are often advised to receive adjuvant treatments (e.g., radiotherapy or chemotherapy) post‐surgery to eradicate the residual tumors due to lower chances of recurrence and prolonged survival. PTT might serve as an alternative adjuvant treatment in better controlling the post‐surgical tumors because of: 1) lack of ionizing radiation that is often harmful for surrounding normal tissues; 2) targeted localization of PTT agents (i.e., nanoparticles) that limits the heating of the tumor tissues; and 3) feasible and accurate delivery of the light to any desired location within the tumors, with minimal damage to adjacent organs or tissues.

**Figure 6:**
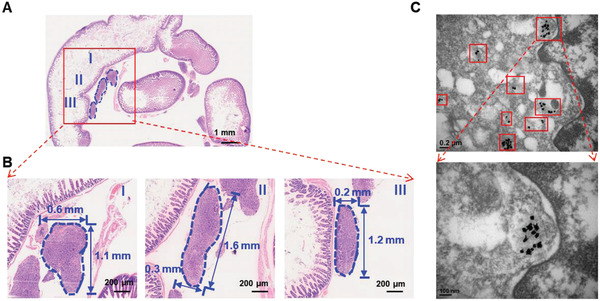
The H&E staining and TEM images of the tissue from post‐surgery site of orthotopic SKOV3 tumor model. A) The low magnification microscope photo of the tissue from the surgery site. B) The high magnification microscope photo of tumor regions from (A), the blue dash line outlined the residue micro‐tumors of the tissue post‐surgery. C) TEM images of the tissues, which showed presence of nanoparticles (red boxes) in post‐operative tumor residues.

**Figure 7:**
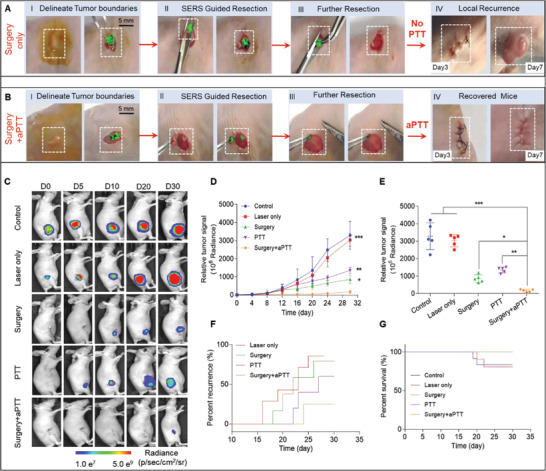
Raman image‐guided resection and adjuvant photothermal therapy (aPTT) of the subcutaneous SKOV3 ovarian tumors. A,B) Raman image‐guided surgery and with the adding of adjuvant photothermal treatment. (785 nm, 150 mW, 5× objective, 0.2 s integration time, and 3–10 min in total). C) Bioluminescent images of the representative mice from 5 different treatment groups. D) Variation of the tumor BLI signals after different treatments. E) Relative tumor signals measured in different groups after 30 days. F) The rate of tumor recurrences in different treatment groups. G) Kaplan–Meier plot showing the rate of mice survival in different groups. In vivo bioluminescence images were used to track tumor signals in different groups of mice. Data are presented as the mean ± SD, *p* values were calculated by Student's *t*‐test, (*n* = 5, **p < 0.01, ***p < 0.001, relative to the control mice).

**Figure 8:**
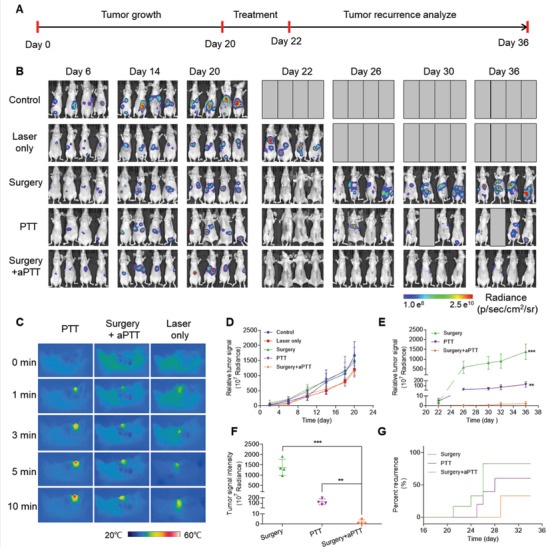
Raman image‐guided resection and adjuvant photothermal therapy (aPTT) of the orthotopic SKOV3 ovarian tumors. A) Timeline of the procedures, including image‐guided surgery in combination with photothermal therapy of the orthotopic SKOV3 ovarian tumor‐bearing mice. B) Bioluminescent images of the mice showing the variation of tumor signals at different treatment stages in all five groups. C) Thermal images of the tumor or tumor bed during PTT, aPTT, and Laser only treatments, representing the thermal response of the nanoparticles or tumor tissue to laser (808 nm, 1.5 W cm^−2^, 10 min). D) Diagram showing variation of the tumor BLI signals in all 5 groups before starting the treatments. E) Variation of the tumor BLI signals during different treatments, started at day 22 and terminated at day 36 (see the timeline in (A) for details). F) Relative tumor signals measured in different groups after 36 days. G) Kaplan–Meier plot showing the rate of tumor recurrences in five different treatment groups. In vivo bioluminescence images were used to track tumor recurrence in different groups of mice. Data are presented as the mean ± SD, *p* values were calculated by Student's *t*‐test, (*n* = 4, **p <0.01, ***p <0.001, relative to the control mice).

In this study, the use of Raman imaging with short exposure time for intraoperative resection of the tumor tissues indicate that Raman image‐guided surgery could facilitate removal of majority of the tumor tissues in a relatively short time, but micro‐tumors are undetectable by Raman imaging with short exposure time (<1 s). Increasing the exposure time helps to detect but is not practically doable due to extremely long scanning time. The H&E staining of the tissues obtained from the post‐surgery region (**Figure** **6**A,B) proved the existence of residual micro‐tumors and the diameter of the tumors are ≈1 mm, which in turn lead to tumor recurrence. It is worth noting that through TEM images of the tissues that were presented above in Figure 6C, the existence of Au‐Ur@DTTC nanoparticles was found in the above micro‐tumor tissues simultaneously. Evaluation of photothermal effect of our Au‐Ur@DTTC nanoparticles revealed that these nanoparticles could be used as photothermal destruction agents (Figure S11, Supporting Information). Moreover, as shown in Figure S12, Supporting Information, ≈80% and ≈50% of the cells were found dead after PTT at 808 nm laser, when they were covered with 2‐ and 4‐mm thick tissues, respectively, indicating the depth‐dependency and efficacy of this procedure in orthotopic tumor models. Hence, a sparse distribution of nanoparticles in residual micro‐tumors was undetectable during the fast‐scan intraoperative Raman imaging, but their photothermal effect improved the survival rate by decreasing the recurrence rate, as shown in **Figures** **7**F and **8**G. The results showed that the PTT approach was effective and selective for tumor areas containing gold nanoparticles.

Following the preliminary PTT tests discussed above, the anti‐tumor efficacy of fast Raman image‐guided surgery with or without adjuvant PTT (aPTT) on subcutaneous and orthotopic ovarian (SKOV3) tumors was further evaluated. As shown in Figure 7A,B, the subcutaneous SKOV3 ovarian tumor‐bearing mice (*n* = 5) were first resected successfully under Raman imaging guidance, and then the mice in the surgery only group (Figure 7A) were sutured, showing tumor recurrence at 7‐days post‐surgery, while no tumor recurrence was fond in the mice in surgery + aPTT group (Figure 7B), with the addition of adjuvant PTT after surgery. To investigate the anti‐tumor efficacy and feasibility of Au‐Ur@DTTC mediated Raman imaging for surgery and/or PTT systematically, a subcutaneous SKOV3 (Luc^+^) tumor‐bearing mice were randomly assigned to 5 treatment groups (three mice per group): 1) Control, 2) Laser only, 3) Surgery (surgery only), 4) PTT (photothermal therapy only), and 5) Surgery + aPTT (combined surgery/adjuvant photothermal therapy). The representative bioluminescence images and corresponding signal intensities of the mice at different time points are displayed after treatment initiation (Figure 7C,D). The relative signal intensities of the tumor (Figure 7E) observed in surgery + aPTT group were significantly lower than other groups on day 30 of post‐treatment (surgery + aPTT versus PTT: **p < 0.01; surgery + aPTT versus surgery: *p < 0.05), suggesting that the aPTT could improve the treatment outcome after surgery. The local recurrences (Figure 7F) among mice undergoing combined surgery + aPTT was significantly lower than those treated only with PTT or surgery, which followed a relatively higher survival rate (Figure 7G).

Also, the effect of Raman image‐guided surgery by adding adjuvant PTT was also investigated by using orthotopic SKOV3 tumor model, which was closer to the real situation of tumor than subcutaneous tumor model. Mice bearing orthotopic SKOV3 tumors were divided into five groups with various treatments (Control, Laser alone, PTT, Surgery, and Surgery + aPTT), and the treatment schedule was shown in Figure 8A. The bioluminescence images (Figure 8B, left) and signals (Figure 8D) of the tumors from all the five groups were similar in all the mice before initiation of the treatments (*p* > 0.1) on day 20. Imaging with a near infrared camera (Figure 8C and Figure S13, Supporting Information) revealed that the surface temperature of the tumor treated with PTT reached 49.5 °C, the surface temperature of the tumor irradiated with laser only was shown at 37.8 °C, and the surface temperature of surgical incision was shown at 44.5 °C, which was lower than tumors of PTT only group. This indicated that only the nanoparticles accumulated in the tumor lesions could generate sufficient heat for tumor ablation (usually >42 °C).^[^ ^48^ ^]^ Since the bulk of tumors were first resected in surgery + aPTT group, the total amount of tumor‐accumulated Au‐Ur@DTTC nanoparticles were less when compared to those treated with only PTT. This lower ablation temperature provided enough thermal energy to kill any residual cancer cells, further reducing the risk of burns in normal tissues surrounding the surgical incision. As expected, the responses to treatment and survival improvements were similar to mice with subcutaneous tumors. Mice treated by surgery or PTT only exhibited continuous tumor growth on post‐treatment day, which was mainly caused by inaccurate resection and the existence of residual tumor cells. Comparatively, the tumor growth rate of mice treated with combined surgery/aPTT showed significant reduction during the 36‐day monitoring period (Figure 8E). The tumor bioluminescence signals on day 36 were significantly lower among the mice treated with surgery + aPTT when compared to those receiving only PTT or surgery treatments (**p < 0.01 for PTT alone group and ***p < 0.001 for surgery alone group, Figures 8F). The recurrence rate of mice treated with surgery + aPTT was 25% only during 36 days, which was mainly due to Au‐Ur@DTTC‐induced adjuvant PTT treatment that ablated the residual micro‐tumors; and mice treated with PTT showed initiation of tumor recurrence on day 25 in 60% of the mice, and 85% of the mice in the surgery group on day 26 (Figure 8G). In addition, the survival rate of the mice in surgery + aPTT group was shown to be improved to 75% when compared to those in control group (Figure S14, Supporting Information). Therefore, the combination of surgery with aPTT could effectively inhibit tumor recurrence due to precise elimination of residual tumor margins without heating side‐effects on adjacent normal tissues.

### 2.9 Hand‐Held Raman Scanning Guided Tumor Surgery

These results showed that Raman imaging using Au‐Ur@DTTC nanoparticles have enabled us to delineate tumor margins and increase the precision of tumor surgeries (Figures 5 and 7A). However, imaging with Raman microscope requires acquisition times ranging from several minutes to hours, depending on the desired resolution and region of interest, limiting the clinical translatability of the technique. We herein used a hand‐held Raman probe as a real‐time intraoperative scanning tool for detecting the tumor tissues based on SERS contrast generated from Au‐Ur@DTTC nanoparticles. This setup enabled faster scanning of the whole surgical area, facilitating real‐time detection of the tumor and tumor margins during resection surgeries. Additionally, these measurements showed that the probe was highly sensitive, and capable of detecting Au‐Ur@DTTC nanoparticles with concentrations as low as 0.1 OD (Figure S15, Supporting Information). Several hand‐held Raman scanners are available commercially, and the one that is already in clinical trials was chosen.^[^ ^49^ ^]^ Photographs that demonstrated this intraoperative setup for subcutaneous SKOV3 tumor surgery were shown in Figure S16A,B, Supporting Information. The Raman spectra collected were collected from the tumors for guiding resection, and the Raman signal intensities were diminished at the end of resection (*n* = 3, Figure S16C, Supporting Information), while these preliminary results are promising, and more studies are needed to evaluate this system using systematic survival studies. Such hand‐held and portable Raman detectors can expedite the translation of Raman image‐guided tumor surgeries to the clinics because of their much shorter data acquisition times (0.2–1 s for one‐point detection) and ease of handling. Also, the addition of aPTT modality to these hand‐held detectors was convenient and can expand the application of this approach to simultaneous Raman imaging‐based surgery and PTT in the near future.

### 2.10 Systemic Toxicity

To assess potential safety and cellular uptake of Au‐Ur@DTTC nanoparticles for conducting in vivo studies, in vitro cytotoxicity was measured by using 3‐(4,5‐dimethylthiazol‐2‐yl)‐2,5‐diphenyl tetrazolium bromide (MTT) assay, and Raman imaging of SKOV3 cells was performed too. The viability concentrations of SKOV3, CT26, HepG2, HEK293, Fibroblasts, and Jurkat T cells incubated with different Au‐Ur@DTTC nanoparticles ranged from 0 to 50 OD (optical density) for 72 h as shown in Figure S17, Supporting Information. The results showed more than 90% cell viability for increased concentrations of Au‐Ur@DTTC nanoparticles, reflecting their minimal cell cytotoxicity and favorable cytocompatibility. In the meantime, prominent SERS characteristics of the prepared Au‐Ur@DTTC nanoparticles have enabled effective Raman imaging of SKOV3 cancer cells after incubation with Au‐Ur@DTTC nanoparticles for 12 h (Figure S18, Supporting Information).

Furthermore, the systemic in vivo toxicity of the Au‐Ur@DTTC nanoparticles was evaluated. Twenty healthy female Balb/c mice (6‐weeks‐old) were randomly divided into 2 groups (*n* = 10 per group) and subjected to different conditions, which were as follows: 1) control group with intravenous injection of PBS (200 µL), and 2) experimental group with intravenous injection of Au‐Ur@DTTC nanoparticles (50 OD, 200 µL). Blood biochemical, hematological, and histological analyses were performed at 2‐weeks and 3‐months post‐injection. Various blood analyses, including albumin, total protein, aspartate transaminase, alanine transaminase, blood urea nitrogen and creatinine were examined (Figure S19A, Supporting Information). The results showed no statistically significant differences (*p* > 0.05) from the experimental group at both time points and similar results were found in evaluating the biochemical performance (Figure S19B, Supporting Information). Corresponding histological sections of the major organs including liver, spleen, kidney, heart, and lung were also evaluated by immunohistochemistry using H&E staining (Figure S19C, Supporting Information). No remarkable toxicity signs were observed at 2‐weeks and 3‐months post‐injection.

Toxicity studies have suggested a relative safety of Au‐Ur@DTTC nanoparticles for clinical translations. However, future studies are required to address short‐ and long‐term side‐effects and body clearance pathways of gold nanoparticles more accurately.

## 3 Conclusions

In summary, a multifunctional nanoplatform was developed for multimodal bioimaging and treatment, including Raman image‐guided tumor surgery and tumor‐selective PTT. PA and whole‐body PET imaging were used for evaluating the biodistribution of these nanoparticles, and to identify regions of interest within the xenograft tumors for monitoring the treatment. Raman imaging allowed demarcation of tumor margins with high sensitivity and spatial resolution. SERS mediated pre‐operative mapping and intraoperative imaging facilitated accurate Raman‐guided tumor resection owing to prolonged blood circulation and efficient tumor vasculature retention of Au‐Ur@DTTC nanoparticles. The enhanced Raman signals of these nanoparticles enabled us to visualize and remove tumor margin residues that were not visible by the naked eyes. Additionally, this approach can effectively treat the tumor margin residues by nanoparticle‐based PTT. Simultaneous Raman‐guided surgery and aPTT have improved the survival rate by up to 75% and postponed tumor recurrence significantly when compared to the control group. Besides, the Au‐Ur@DTTC nanoparticles were biocompatible, and no overt toxic effects were observed in both the in vitro and in vivo toxicity studies. Also, our technique was couple with a portable hand‐held Raman detector. The preliminary tests showed that this hand‐held Raman detector could be effectively used for real‐time intraoperative Raman guided resection and PTT of different types of tumors in the near future.

Based on the above studies, it is concluded that combination of Raman‐image guided surgery with aPTT has great promise for tumor therapy. That is, without adding aPTT, Raman image‐guided surgery alone is not regarded as a good option, specifically for the management of complex tumors (i.e., ovarian and colon). Despite of ablation of major parts of these residual tumors and decrease of the chance of recurrence and improved survival relatively by post‐operation PTT, the tumor recurrence caused by residual cancer cells that escaped from aPTT cannot be ignored. This situation might be mainly caused by uneven distribution of laser beam for PTT, which was followed by uneven PTT for residual tumors, especially with the use of beam expander to expand the spot diameter. Moreover, the limitation to superficial and surgically accessible tumors due to insufficient tissue penetration of the laser beam (4 mm) and detection of Raman signals is another aspect that required attention. While this is important for achieving improved therapeutic efficacy in clinical applications, and the feasibility of the technique for the management of ultra‐small metastatic tumors, and surgically inaccessible tumors should be further investigated in the near future.

## 4 Experimental Section

##### Materials

Gold chloride trihydrate (HAuCl_4_·3H_2_O; ≥99.9% trace metal basis; Sinopharm Chemical Reagent Co., Ltd, Shanghai, China), deionized water (18.2 MΩ cm), sodium hydroxide (NaOH; 98–99.5%; Aladdin, Shanghai, China), hydrogen peroxide (H_2_O_2_; ≈30% (v/v); Aladdin, Shanghai, China), polyvinyl pyrrolidone (PVP; MW 10 000, 40 000, 360 000; Sigma‐Aldrich, St. Louis, MO), hydrochloric acid (Sinopharm Chemical Reagent Co., Ltd, Shanghai, China), nitric acid (HNO_3_; AR; Sinopharm Chemical Reagent Co., Ltd, Shanghai, China), ethyl alcohol (C_2_H_6_O; AR; Sinopharm Chemical Reagent Co., Ltd, Shanghai, China), and near‐infrared‐dye 3,3′‐diethylthiatricarbocyanine iodide (DTTC; 99%; Sigma‐Aldrich, St. Louis, MO) were purchased.

##### Synthesis of Au‐Ur

Au‐Ur samples were prepared at room temperature by following the green chemistry processes, and all the glassware were washed with aqua regia, ethanol, and deionized water before reaction. A total of 200 µL of 0.1 m HAuCl_4_ aqueous solution was added to a glass tube with 20 mL PVP aqueous solution. Next, 1 mL of 30 vol% aqueous solutions of H_2_O_2_ was added after vigorous stirring of the solution for 30 s. The solution was then adjusted to pH 11 by addition of NaOH (1 m). During the addition, the color of the solution changes from transparent to turbid blue and Au‐Ur solution was then obtained. The Au‐Ur nanoparticles were collected by centrifugation (3763 g for 15 min at 26 °C) and redispersed in deionized water to produce a colloidal suspension that can be used for subsequent experiments (Final Au concentration = 2 mg mL^−1^).

##### Synthesis of DTTC Tagged SERS Nanoparticles (Au‐Ur@DTTC)

DTTC (5 × 10^−6^ m) solutions with varying volumes ranging from 1 to 10 mL were added to 1 mL of the above collected colloidal Au‐Ur solutions by stirring at medium speed (2.35 g). The mixtures were then incubated for 20 min at 28–30 °C. The volume ratio of DTTC and gold nanoparticles were optimized to obtain maximum SERS intensities and minimum colloidal aggregation. Next, the Au‐Ur@DTTC nanoparticles solution was purified by three rounds of centrifugation at 3763 g for 15 min at 26 °C. PVP (0.1 mg mL^−1^) was added to the solution that was prepared as a stabilizer to improve the stability of Au‐Ur@DTTC nanoparticles. The resulting DTTC tagged Au nano‐urchin (Au‐Ur@DTTC) was redispersed in PBS buffer for conducting animal studies.

##### Characterization of Nanoparticles

TEM and EDS (JEM‐2100F, 200 kV, JEOL, Inc. Japan) were used for investigating the structural morphology of the nanoparticles. SEM images were obtained by an S‐4800 field emission scanning electron microscope (Hitichi, Inc. Japan). Size distribution and zeta potential of the nanoparticles were measured using the DLS system (Zetasizer Nano‐ZSE, Malvern Instruments, UK). The UV–vis absorption spectra were characterized by UV‐2600 spectrophotometer (Shimadzu, Inc. Japan). The UV–vis spectrophotometer was able to measure when the concentration of Au‐Ur@DTTC nanoparticles was in the range of 0–2 optical density. Therefore, the nanoparticle solutions were diluted for 25–30 times prior to recording their UV–vis spectra. XRD patterns were obtained on a D8 Advance (Bruker, Germany) diffractometer with Cu‐K*α* radiation at *λ* = 0.154 nm. A PHI Quantera SXM XPS system (Ulvac‐PHI, INC, Japan) was used for XPS. Finally, the Raman‐related tests were investigated by Renishaw InVia microscopy system equipped with a 785 nm laser (Renishaw Inc., UK). All samples were first purified by two centrifugation steps at 4248 g for 10 min and redispersed in PBS to remove any excess reagents before these measurements were obtained.

##### Cell Culture

To enable in vivo bioluminescence imaging, firefly luciferase‐transfected CT26, SKOV3 and melanoma (A375M) cells were utilized. The cells were transfected with the plasmid (pLenti CMV‐LUC‐Puro) using transfection reagent Lipofectamine 2000 (Thermo‐fisher technology Co., LTD, Shanghai, China). After 48 h, the cells were treated with puromycin (0.5 µg mL^−1^) to select the transfected cells and generate stable cell lines. These cells were subjected to luciferase assay (data not shown) to confirm luciferase activities. For cytoxicity analysis, both cancer cells (SKOV3, CT26, and HepG2) and normal cells (HEK293, Fibroblats, and Jurkat T) were used. The cell lines were grown in complete DMEM with 10% fetal bovine serum and 1% Penicillin–Streptomycin solution according to the given protocol and then incubated at 37 °C with 5% CO_2_, followed by detachment with EDTA for 3 min and collecting after reaching ≈80% confluency for in vitro and in vivo experiments.

##### Animal Studies

All animal studies were performed in accordance to the requirements of Zhejiang University Institutional Animal Care and Use Committee (IACUC) for care and use of laboratory animals in research. 6–8 weeks old female Balb/c nude mice were obtained from Slac Laboratory Animal Co., Ltd (ShangHai, China) and Charles River (Wilmington, MA). CT26 and SKOV3 tumor cells (Luc^+^, 1 × 10^6^ cells in 150 µL of PBS) were subcutaneously injected into each mouse to establish xenograft models. The orthotopic CT26 and SKOV3 mice models were established by injecting 5 × 10^5^ CT26 and SKOV3 cells intraperitoneally into a volume of 50 µL PBS. Tumor progression was monitored by using bioluminescent imaging carried out with an IVIS spectrum system (PerkinElmer, Waltham, MA) after intraperitoneal injection of d‐luciferin (150 mg kg^−1^ of mouse body weight) in the subsequent weeks. All mice in the experiments underwent humanitarian treatment, and 15 day‐post treatment was selected as the experimental endpoint, and this was because the volume of the tumors was very big and some mice even show severe ascites at that point.

##### Imaging Procedures

1 Imaging of NPs in solutions. A Raman spectrometer (Renishaw) equipped with a 300 mW 785 nm laser and a 1‐inch charge‐coupled device detector was used for Raman imaging. To determine Raman spectra of different compounds and nanoparticles, 1 mL of pure DTTC (1 µm), Au‐Ur (0.15 mg mL^−1^), and Au‐Ur@DTTC nanoparticles (0.15 mg mL^−1^) were dispersed in 1 mL of PBS, and their Raman spectra were acquired at 785 nm laser (150 mW laser power, 150 mW, 5× objective, 0.5 s integration time).
2 Cell imaging. For evaluating Raman spectra of Au‐Ur@DTTC nanoparticles on cells during in vitro studies, SKOV3 cells in logarithmic phase of growth were cultured in a 96‐well dish for 24 h and then incubated with pure DTTC (1 µm in medium), Au‐Ur (0.15 mg mL^−1^ in medium), and Au‐Ur@DTTC nanoparticles (0.15 mg mL^−1^ in medium) for 12 h. The regions of 96‐well plate (5 × 5 mm) that seeded SKOV3 cells were scanned using Renishaw InVia microscope at 785 nm laser (75 mW), with 0.5 s integration time, 5× objective, step size of 200 µm, and ≈10 min in total. For single cell imaging, the SKOV3 cells were seeded in 6‐well plate and incubated with Au‐Ur@DTTC nanoparticles (0.15 mg mL^−1^ in medium) for 12 h. Raman imaging was then used to capture at 785 nm laser (75 mW) with 0.5 s integration time, step size of 2 µm, 20× objective, and ≈15 min in total. All the cells were washed with PBS for three times before imaging.
3 Animal imaging. 200 µL of Au‐Ur@DTTC nanoparticles (2 mg mL^−1^ in PBS) were injected into mice bearing subcutaneous or orthotopic CT26 and SKOV3 tumors via the tail vein 24 h prior to SERS imaging. After a circulation period of 24 h, the mice were intraperitoneally injected with 3 mg d‐luciferin in 200 µL of PBS for BLI and evaluation of tumor growth. When the tumor bioluminescence signal intensity was substantially strong (ROI values ≥ 5 × 10^7^ photons s^−1^ cm^−2^ sr^−1^, 20 min after d‐luciferin injection), the mice (*n* = 6) were anesthetized by isoflurane (2–3%), and the peritoneal cavity was exposed by a sagittal incision. For the BLI of whole abdomen, the mice were imaged with IVIS spectrum imaging system (PerkinElmer, Waltham, MA) within 20 min. For SERS mapping, the anesthetized mice were transferred to the Raman spectrometer (Renishaw, UK) of 785 nm laser, which involves a relatively higher tissue penetration (i.e., 4 mm) and lower absorbance by the biological tissues. The StreamLine acquisition mode was used for Raman scanning, in which multiple spectra were acquired under continuous laser illumination, in which the microscope stage moves in straight lines constantly. The spatial resolution of the scan was set to 14.2 µm along the lines and 200 µm across. All the Raman mapping images were scanned ex vivo using the laser power of 150 mW, 5 × objective and 0.2 s of integration time. The total imaging time depends on the scanning area. The scanned areas in the in vivo Raman imaging experiments ranged from 4 to 300 mm^2^, resulting in a total imaging time of 0.5 to 35 min. For experiments based on sequential administration of Au‐Ur@DTTC nanoparticles and ICG, the mice were intravenously injected with Au‐Ur@DTTC nanoparticles followed by intravenous injection of ICG (0.5 mg mL^−1^) after 25 min. The mice were anesthetized with 2–3% isoflurane for conducting fluorescence imaging after 24 h of these injections, and the images were captured using IVIS spectrum imaging system (excitation at 745 nm and emission at 810 nm). Raman imaging of the mice abdominal area was performed after in situ and ex vivo fluorescent imaging. Upon completion of imaging, the tissues of interest were fixed in 4% paraformaldehyde (PFA) and sliced into 3–5 mm thick blocks. The blocks were scanned again prior to the embedment of paraffin and stained with H&E for histopathological analyses using 15 µm tissue sections.

##### Efficacy Evaluation of Photothermal Response of Au‐Ur@DTTC and Spherical Au@Silica Nanoparticles

To evaluate the photothermal response of Au‐Ur@DTTC nanoparticles, 0.5 mL solution of these nanoparticles were transferred into a quartz cuvette and the temperature was measured every minute (until 10 min) using a digital thermometer that was connected to a thermocouple device. The top of the cuvette was sealed with a cap to reduce heat loss and evaporation from the surface of the solution.

To evaluate the in vitro cell killing caused due to nanoparticles photothermal effect, the SKOV3 cells (10000 cells per well of a 96‐well plate) were incubated with Au‐Ur@DTTC (0.15 mg mL^−1^) for 24 h. PTT was performed under 808 nm laser (MDL‐N‐808‐10W, Changchun, China) for 5 min (spot size 5 mm, 0–1.5 W cm^−2^). The penetration depth tests were conducted by covering different thicknesses of chicken tissues (0, 2, 4, and 6 mm) on 96‐well plate. Following these treatments, cell viability was performed by MTT assay kit (YEASEN, Shanghai, China). Live/dead cells were stained using a Calcein‐AM/PI double staining kit (YEASEN, Shanghai, China), and imaged with fluorescence microscopy (20× magnification). The measurements were taken thrice 24 h post laser irradiation (*n* = 3).

##### Intraoperative Raman Imaging and aPTT

The mice bearing subcutaneous or orthotopic SKOV3 tumors were intravenously injected with Au‐Ur@DTTC nanoparticles (200 µL, 2 mg mL^−1^). After 24 h, surgeries were performed to remove the primary tumor based on fast Raman imaging with an InVia Raman microscope (Renishaw) equipped with a 785 nm diode laser. Subsequently, the surgical bed (1 cm × 2 cm) was irradiated using an 808 nm laser (1.5 W cm^−2^) for 10 min. After treatment and suturing of the mice, tumor recurrence was monitored by BLI.

##### Evaluation of In Vivo Toxicity

Twenty healthy mice (8 weeks old Balb/c mice) were used to investigate the toxicity of Au‐Ur@DTTC nanoparticles. The mice were divided into two groups (*n* = 10), and then were injected via tail vein with Au‐Ur@DTTC nanoparticles (20 mg kg^−1^, 200 µL per mouse) and PBS (200 µL per mouse), respectively. The blood samples were obtained by submandibular bleeding (500 µL) for 2 weeks (*n* = 5) or 3 months (*n* = 5) after injecting nanoparticles and were used for blood chemistry and complete blood panel analyses. Meanwhile, the major organs (such as liver, spleen, kidney, heart, and lung) were harvested from the necropsy of histological analyses. The organs were fixed in 4% PFA, embedded in paraffin, sectioned (15 µm), and finally stained with H&E). The sections were scanned under a 20× inverted optical microscope (Olympus, Japan).

##### Statistical Analysis

All data were presented as means ± standard deviation (mean ± SD) and were compared by means of an unpaired, two‐tailed Student's *t*‐test (two groups) or one‐way ANOVA with Tukey post‐hoc test (multiple groups). Sample sizes (n) were mentioned on each figure legend. All the statistical analyses were conducted by using GraphPad Prism software (version 8.0). Asterisks indicated significant differences (*p < 0.05, **p < 0.01, ***p < 0.001, and ****p < 0.0001). All animal studies were performed after randomization.

## Conflict of Interest

The authors declare no conflict of interest.

## Supporting information

Supporting Information
