# Supplementary material for: Intraoperative Assessment and Photothermal Ablation of the Tumor Margins Using Gold Nanoparticles
Source: Adv Sci (Weinh). 2021 Jan 18;8(5):2002788. doi: 10.1002/advs.202002788 (PMC7927626; doi:10.1002/advs.202002788)
Supplement: Supplementary file 1 — Supporting Information [file ADVS-8-2002788-s001.pdf]

© 2020 Wiley-VCH GmbH

ADVANCED SCIENCE
Open Access

Supporting Information

for *Adv. Sci.,* DOI: 10.1002/advs.202002788

Intraoperative Assessment and Photothermal Ablation of the Tumor Margins Using Gold Nanoparticles

Qiaolin Wei, Hamed Arami, Hélder A. Santos,

Hongbo Zhang,Yangyang Li,Jian He, Danni Zhong, Daishun Ling, and Min Zhou*

Supporting Information

Intraoperative Assessment and Photothermal Ablation of the Tumor Margins Using Gold Nanoparticles

Qiaolin Wei,[1,2,3,†] Hamed Arami,[4,†] Hélder A. Santos,[5,6,†] Hongbo Zhang,[7,†] Yangyang Li,[2,†] Jian He,[2] Danni Zhong,[2] Daishun Ling,[8] and Min Zhou[1,2,3,9,*]

[1]The Fourth Affiliated Hospital of Zhejiang University, School of Medicine, Zhejiang University, Hangzhou, 310009, P.R. China

[2]Institute of Translational Medicine, Zhejiang University, Hangzhou, 310009, P.R. China

[3]State Key Laboratory of Modern Optical Instrumentations, Zhejiang University, Hangzhou, 310058, P.R. China

[4]Molecular Imaging Program at Stanford, Department of Radiology, Stanford University, Stanford, CA 94305–5427, USA

[5]Drug Research Program, Division of Pharmaceutical Chemistry and Technology, Faculty of Pharmacy, University of Helsinki, Helsinki, FI-00014, Finland

[6]Helsinki Institute of Life Science (HiLIFE), University of Helsinki, FI-00014, Helsinki, Finland

[7]Pharmaceutical Science Laboratory, Åbo Akademi University, Turku, 20520, Finland

[8]Institute of Pharmaceutics, College of Pharmaceutical Sciences, Zhejiang University, Hangzhou, Zhejiang 310058, P.R. China

[9]Key Laboratory of Cancer Prevention and Intervention, National Ministry of Education Zhejiang University, Hangzhou, 310009, P.R. China

[†]These authors contributed equally to this work

*Corresponding authors.

E-mail: zhoum@zju.edu.cn (M.Z.)

1. **Experimental Section**
2. **Figure S1-S20**

Experimental Section

Evaluation of the *in vitro* cytotoxicity

The *in vitro* cytotoxicity of Au-Ur@DTTC NPs was measured by MTT (3-(4,5-dimethylthiazol-2-yl)-2,5-diphenyltetrazolium bromide, Signal-Aldrich) assay. SKOV3 and CT26 cells were seeded in the 96-well plates at a density of 5 $x10^3$ cells per well in complete medium (DMEM containing 10% FBS and 1% antibiotics (100 U/mL penicillin and 100 μg/mL streptomycin)) at 37 °C and under a humidified atmosphere containing 5% CO_2. Cells without treatment were used as control. After 48 h, 10 uL of MTT solution (5 mg/mL) was added to each well and incubated for an additional 4 h at 37 °C for MTT formazan formation. Then, the media were completely removed, and 200 μL DMSO was added to each well to dissolve MTT formazan at 37 °C. The plates were gently shaken for 5 min to ensure the dissolution of formazan. The absorbance values were measured at 570 nm wavelength, using a microplate reader (Infinite F200, TECAN, Austria). Triplicates were used for each condition.

In vivo photoacoustic imaging (PAI)

Mice bearing SKOV3 tumors (n = 3) were *i.v.* injected with Au-Ur@DTTC NPs (2 OD) and imaged using a PA system with an 808-nm laser as the excitation source. For *in vivo* photoacoustic imaging, BALB/c nude female mice (6-8 weeks, purchased from SLAC, Shanghai, China) with subcutaneous tumor xenografts (n = 3) were anesthetized with 2.5% isoflurane delivered via a nose cone, and then Au-Ur@DTTC NPs (200 μL) was injected to each mouse *via* the tail vein. After this injection, mice were scanned at different time post-injection times (1 h, 5 h, and 24 h, MSOT inVision 128, iThera medical, Germany) to record the PA images under 808 nm laser excitation,

consuming 5 mins for each time point. Anesthetized animals were placed in supine position and PA images were acquired with an in-plane resolution of approximately 150 μm. Linear-mode-based reconstruction and linear regression spectral were applied using ViewMSOT (iThera Medical).

Radiolabeling of [^{64}Cu]Au-Ur@DTTC NPs

For conjugation of radiometal chelator to nanoparticles, DOTA-LA (1 mg/mL, 10 μL) was mixed with 1 mL aqueous solution of Au-Ur@DTTC NPs for 4 h at room temperature. For radiolabeling, aliquots of Au-Ur@DTTC NPs (DOTA) (0.5 mL) in 0.1 mol/L sodium acetate solution (pH 5.5) were mixed with an aqueous solution of $^{64}CuCl_2$ (~1 mCi) at 37 °C for 30 min. The radiolabeled nanoparticles were then purified by centrifugation at 3800 g for 5 min and re-dispersion in PBS (3 times). The radiolabeling efficiency and the stability of labeled nanoparticles were analyzed using a Bioscan IAR-2000 TLC scanner (Washington, DC, USA).

In vivo **pharmacokinetics**

Five Swiss female mice (8 weeks) were injected with [^{64}Cu] Au-Ur@DTTC NPs (0.074 MBq per mouse) *via* tail veins, and blood samples (10 μL) were collected from their retinal vein before and after intravenous injection at different time points (0.25, 0.5, 0.75, 1, 2, 3, 4, 6, 8, and 24 h). The radioactivity of the blood samples was measured by a gamma counter (Packard Cobra, Ramsey, MD, USA). The pharmacokinetic parameters of the blood were analyzed by WinNonlin 5.0.1 software (Pharsight Corporation, Palo Alto, CA), and the decay curves of the copper content in the blood samples were generated and fitted with a two-compartmental model to determine the blood half-life of the nanoparticles.

In vivo **biodistribution**

[^{64}Cu] Au-Ur@DTTC NPs (0.74 MBq per mouse) were injected into female nude mice (8 weeks old, n = 5) with subcutaneous SKOV3 tumors (the volume of the tumors was

300 mm^3) *via* tail veins. Mice were euthanized by CO_2, 24 h after administration of the nanoparticles. Heart, liver, spleen, lung, kidneys, stomach, intestine, muscle, bone, brain, blood, and tumors were collected and weighted, and radioactivity was measured using a gamma counter (Packard Cobra, Ramsey, MD, USA).

Small animal PET/CT Imaging

200 μL of [^{64}Cu] Au-Ur@DTTC NPs (7.4 MBq per mouse) were injected into female nude mice (8 weeks, n = 5) with subcutaneous SKOV3 tumors (the volume of the tumors was 300 mm^3) *via* tail veins. Animals were anesthetized with 2% isoflurane and placed in the prone position, and PET/CT images were acquired 1, 5, and 24 h after injection of the nanoparticles using a high-resolution Inveon μPET/CT R4 scanner (Siemens Preclinical Solutions, Knoxville, TN) for 10 min static acquisition. The images were reconstructed by MAP algorithm, IDL (Ver6.2 Research Systems, Colorado, USA) and ASIPro (Concored Microsystems, Knoxvile, USA) software.

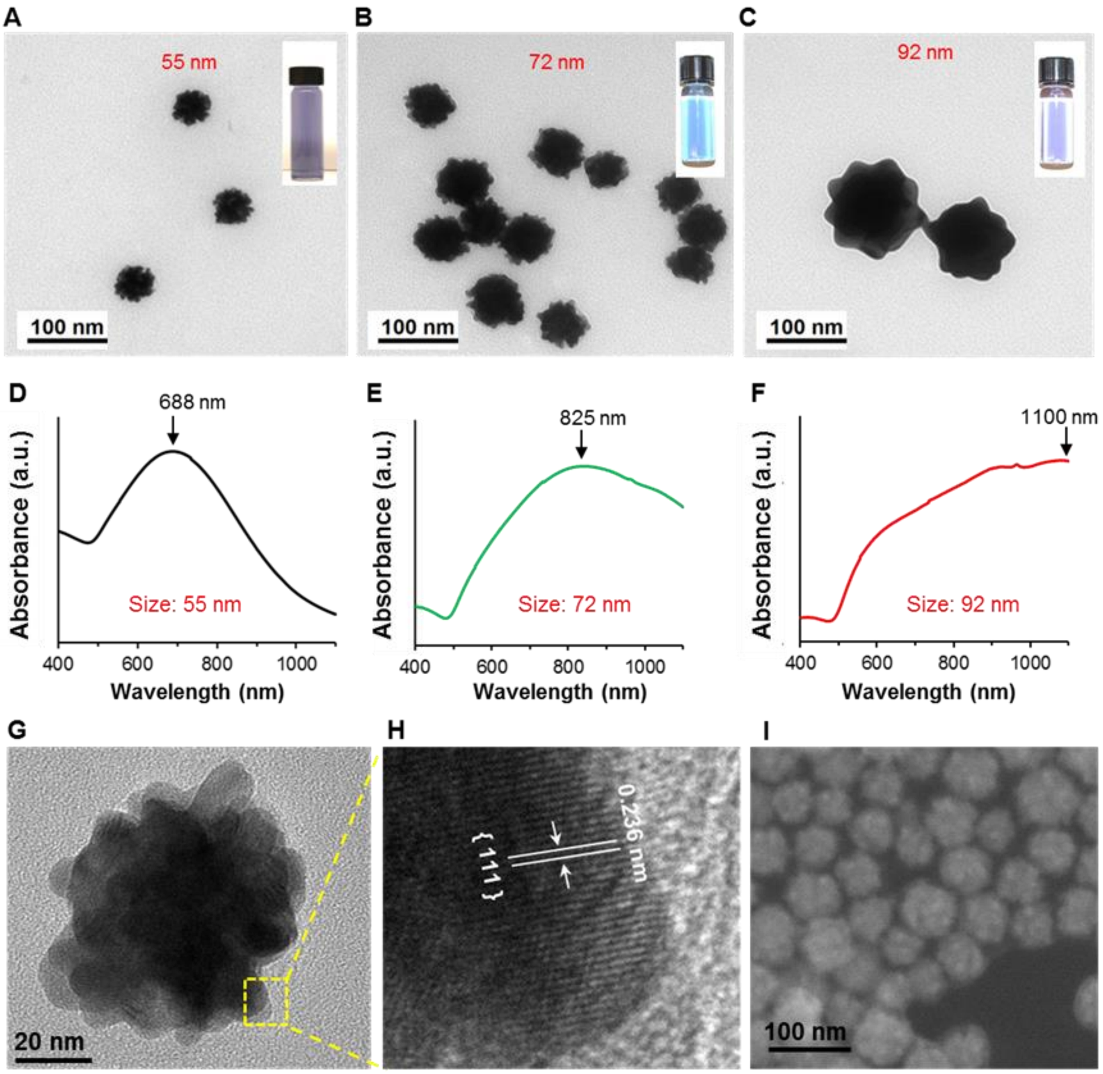

Figure S1. Synthesis and characterization of gold nano-urchins (Au-Ur). (A-C) TEM images of Au-Ur synthesized by addition of 100, 200, and 500 mM sodium hydroxide solutions to gold precursor solution. Insets: photographs of the corresponding nanoparticle solutions. By adjusting the sodium hydroxide concentration, the mean particle diameters were tuned to ~55 nm, ~72 nm, and ~92 nm, respectively. In addition, the color of the three solutions was distinguishable and changed from purple-red to blue, and then to lilac, due to the red-shift characteristic surface plasmon resonance of Au-Ur. (D-F) The UV-vis absorbance spectra of different sizes of the Au-Ur. The absorption peaks were dependent on size of the gold nanoparticles, and larger NPs demonstrated more red-shifts. (G) A TEM image of a single Au-Ur, showing that the Au nanoparticle was branched and formed a nanostructure similar to sea urchins. (H) A high-resolution TEM image of an Au-Ur nanoparticle, showing the lattice fringes of crystalline gold. The distance between the adjacent lattice fringes was 0.236 nm, which was well consistent with the {111} interplanar spacing of cubic Au unit cells. (I) SEM image of the Au-Ur nanoparticles, demonstrating uniform spherical nanoparticles with urchin-like protruding surfaces and an average diameter of ~92 nm.

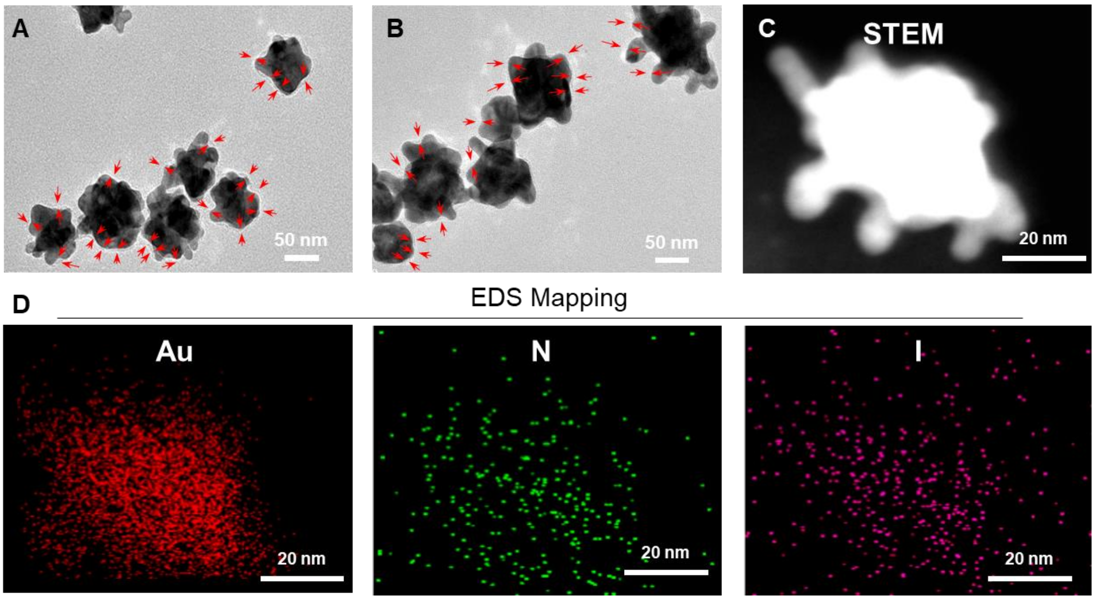

Figure S2. Characterization of Au-Ur@DTTC NPs. (A-B) TEM images of Au-Ur@DTTC NPs. The red arrows denote the DTTC and PVP coating layer (also shown in Fig. 1). (C) STEM image and (D) the corresponding EDS mapping images. Compared with the dense population of red spots that represent gold core, the sparse green dots represent nitrogen atoms in DTTC ($C_{25}H_{25}IN_2S_2$) and PVP [$(C_6H_9NO)_n$] and the signals from iodine element (shown as pink dots on right) are only originated from DTTC ($C_{25}H_{25}IN_2S_2$). The EDS mapping images denote that the DTTC and PVP are uniformly loaded on the surface of Au-Ur nanoparticles.

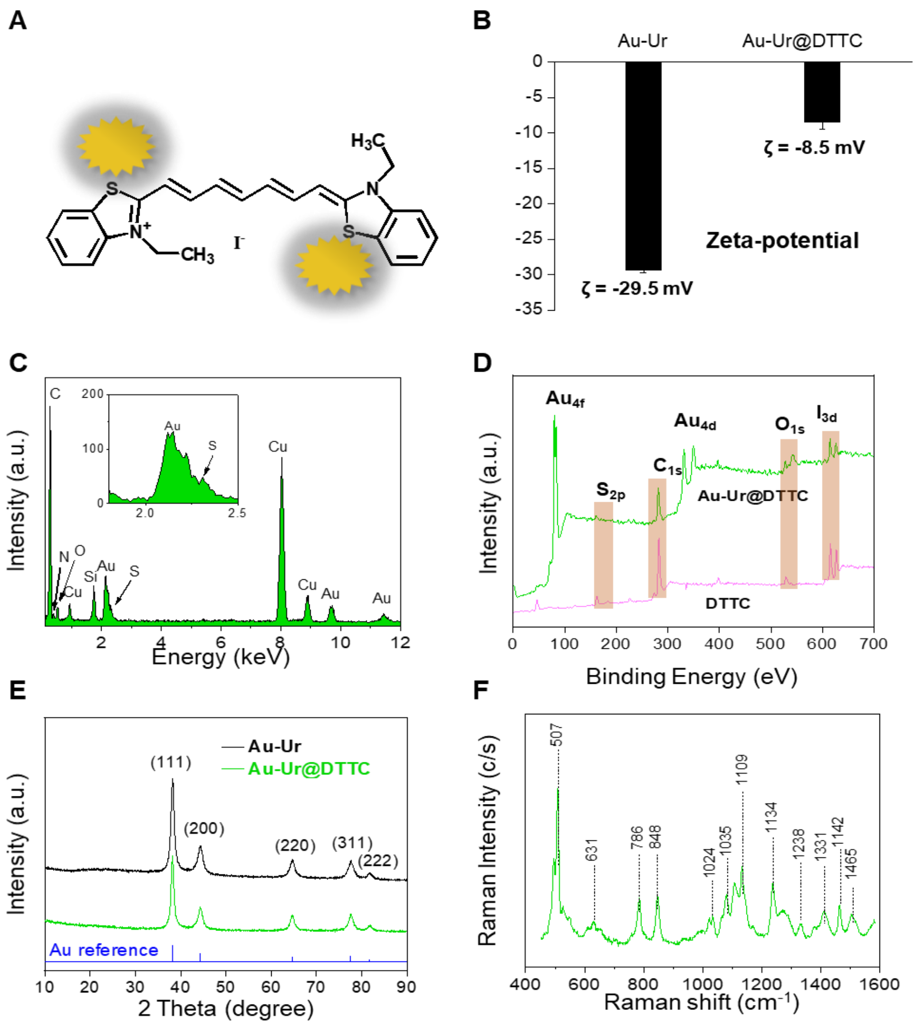

Figure S3. Characterization of Au-Ur@DTTC nanoparticles. (A) Chemical structure of DTTC; (B) Zeta-potential (n = 3), (C) Energy-dispersive X-ray spectroscopy spectrum (EDS), (D) X-ray photoelectron spectroscopy (XPS), (E) X-ray diffraction (XRD) pattern and (F) Raman spectrum of Au-Ur@DTTC nanoparticles. The Zeta potential changed from -29.5 to -8.5 mV, due to presence of DTTC on the surface of the Au-Ur. EDS showed co-existence of Au, C, S, N, and O elements in Au-Ur@DTTC nanoparticles. C, S, N, and O peaks are attributed to the DTTC molecules and confirm successful attachment of DTTC onto Au-Ur. XRD pattern of the nanoparticles showed well-defined diffraction peaks at {111}, {200}, {220}, and {311}, matching well with the fingerprint XRD pattern of cubic Au phase (JCPDS No. 04-0784). XRD results further confirm that the absorbed DTTC molecules did not compromise the crystallinity of the gold nanoparticles. XPS spectra of the synthesized Au-Ur@DTTC nanoparticles and DTTC molecules were recorded to determine the stoichiometry of the elements. The appearance of the characteristic peaks located at ~161, 282, and 615 eV were contributed to S, C, O, and I elements, and confirmed the successful loading of DTTC molecules onto the nanoparticles.

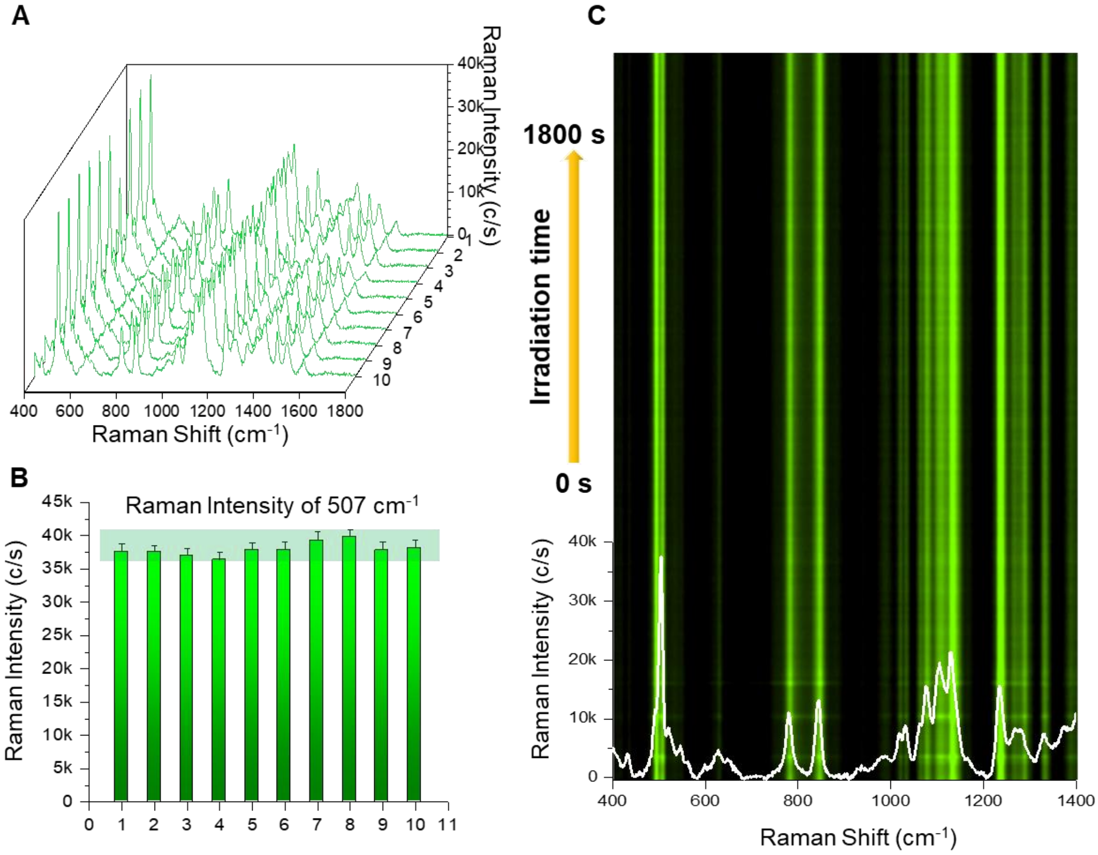

Figure S4. Monitoring the photostability of the Au-Ur@DTTC nanoparticles. (A) Nanoparticles were irradiated by 785 nm laser (150 mW, 0.2 s integration time, 50 × objective) continuously for 30 min and Raman spectra were recorded every 3 min. (B) Raman signal intensities of the 507 cm^{-1} peak. (C) Representative time-resolved Raman signal during 30 min, where the nanoparticles exhibit stationary Raman signal intensity of characteristic Raman bands derived from DTTC.

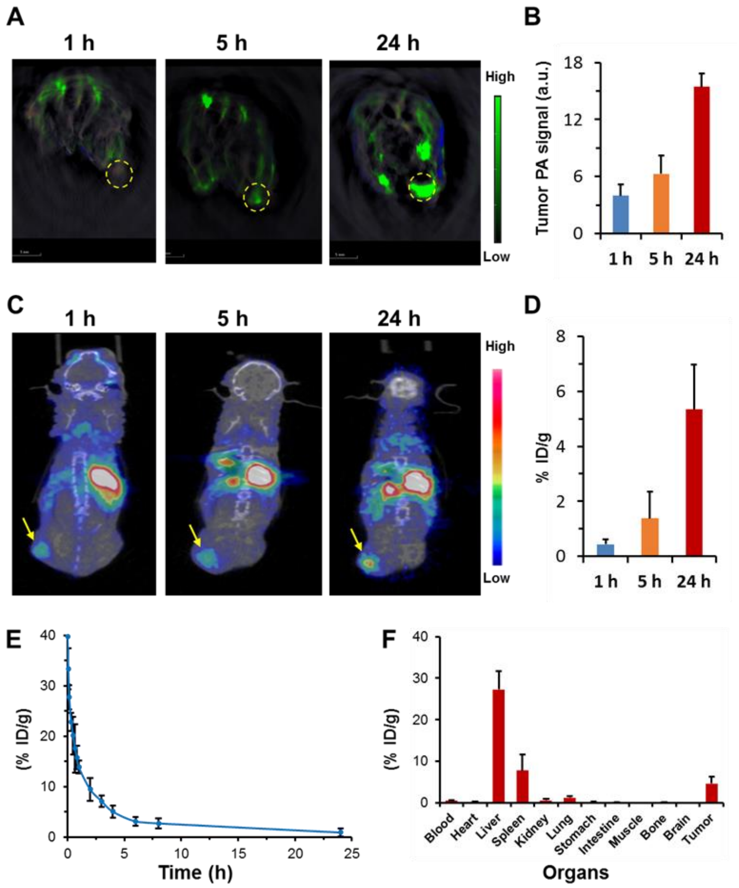

Figure S5. ***In vivo* multimodal imaging and pharmacokinetic performance of the Au-Ur@DTTC nanoparticles.** (A) Photoacoustic images of the mice with SKOV3 tumor, at different Au-Ur post-injection times. The contrast in the tumor (yellow dotted circles) was selectively enhanced using viewMSOT software (iThera Medical). Quantified results in (B) show that the uptake of the nanoparticles by the tumors was increased at 5 and 24h post-injections times. (C) Representative PET images of the mice after intravenous injection of ^{64}Cu-labeled Au-Ur@DTTC nanoparticles, recorded at different post-injection times (n = 3). Tumors are shown with yellow arrows. PET signals within the tumors were measured and are shown in (D). (E) Pharmacokinetics of ^{64}Cu-labeled Au-Ur in Balb/c mice, determined by measuring the ^{64}Cu radioactivity of the blood samples collected at different post-injection times (n = 5). (F) Biodistribution of ^{64}Cu-labeled Au-Ur@DTTC nanoparticles in various organs and tissues of mice 24 h after injection (n = 5).

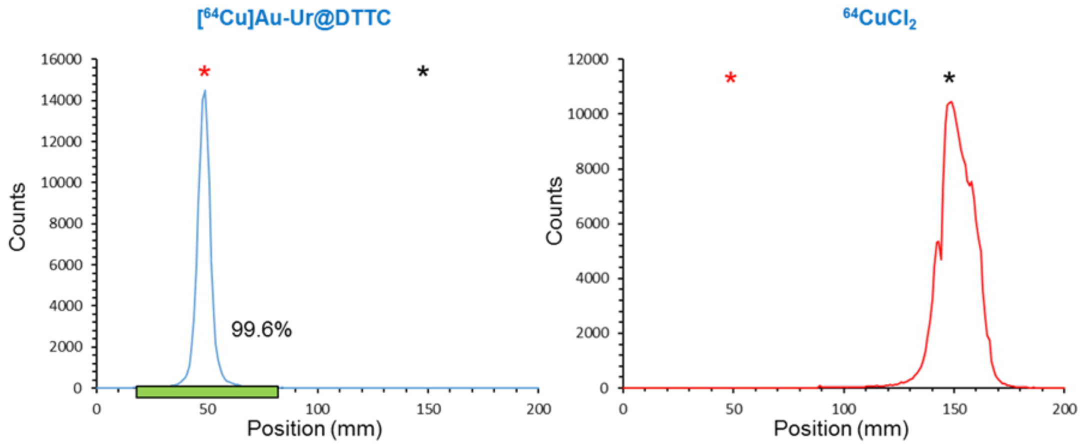

Figure S6. Evaluation of the nanoparticles radiolabeling efficiency. Representative radio-ITLC chromatogram of [^{64}Cu]Au-Ur@DTTC NPs in fetal bovine serum (FBS) at 37ºC at 24 h (n = 3). The red asterisk represents the activity remaining at the origin of the ITLC strip and the black asterisk represents the activity at the solvent front. The whole activity appeared at the origin (*i.e.* where the nanoparticles were located) in the NPs solution, whereas the majority of activity was observed at the solvent front in control samples containing free $^{64}CuCl_2$.

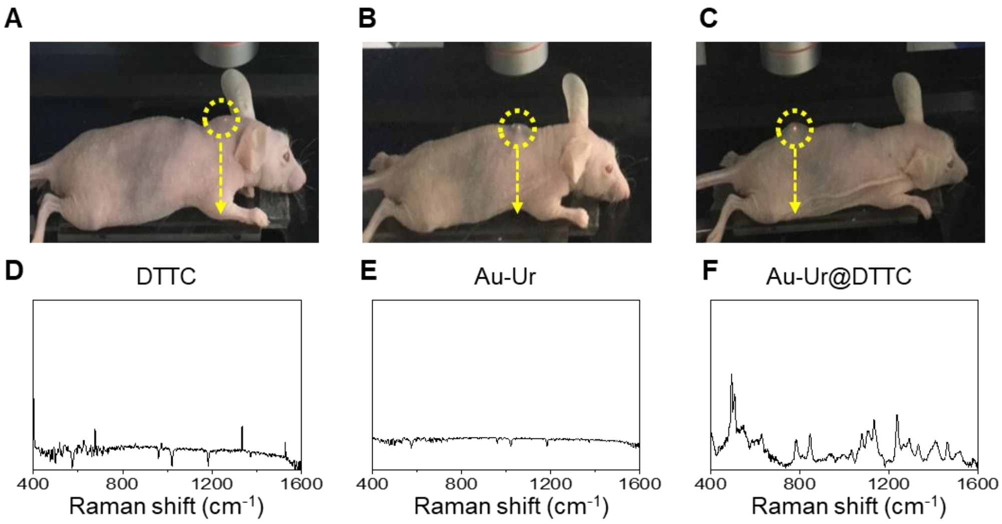

Figure S7. ***In vivo*** **Raman imaging after subcutaneous injection of the nanoparticles into mice.** Photographs illustrating the experimental setup of the Raman spectroscopy and corresponding *in vivo* Raman spectra of a mouse injected subcutaneously with DTTC (A and D), Au-Ur (B and E), and Au-Ur@DTTC NPs (C and F). 200 μL of each sample was injected into each mouse. The concentration of Au-Ur and Au-Ur@DTTC NPs were 2 OD, and the concentration of DTTC was 5 μg/mL.

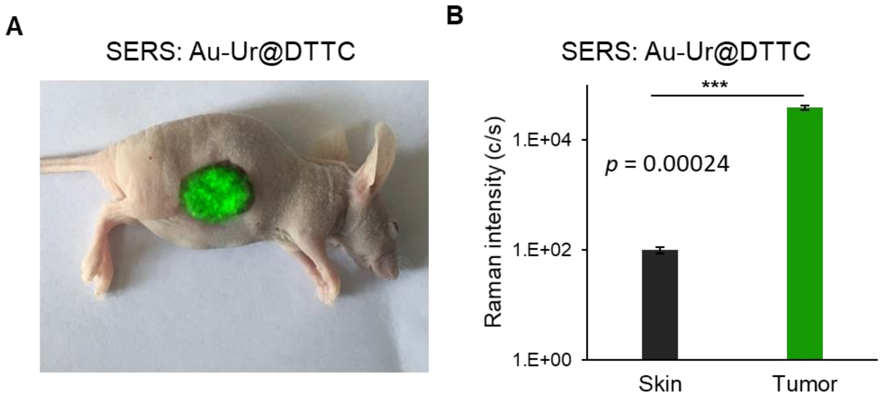

Figure S8. SERS imaging of the tumor and surrounding tissues in mice (n = 3) with subcutaneous SKOV3 tumors. (A) Raman images of tumors overlaid on the photograph of the mouse, and (B) the corresponding Raman signal intensities recorded at the tumor and adjacent skin tissues, p =0.00024 vs. skin. SERS imaging demonstrated a significantly higher signal to noise ratio (SNR).

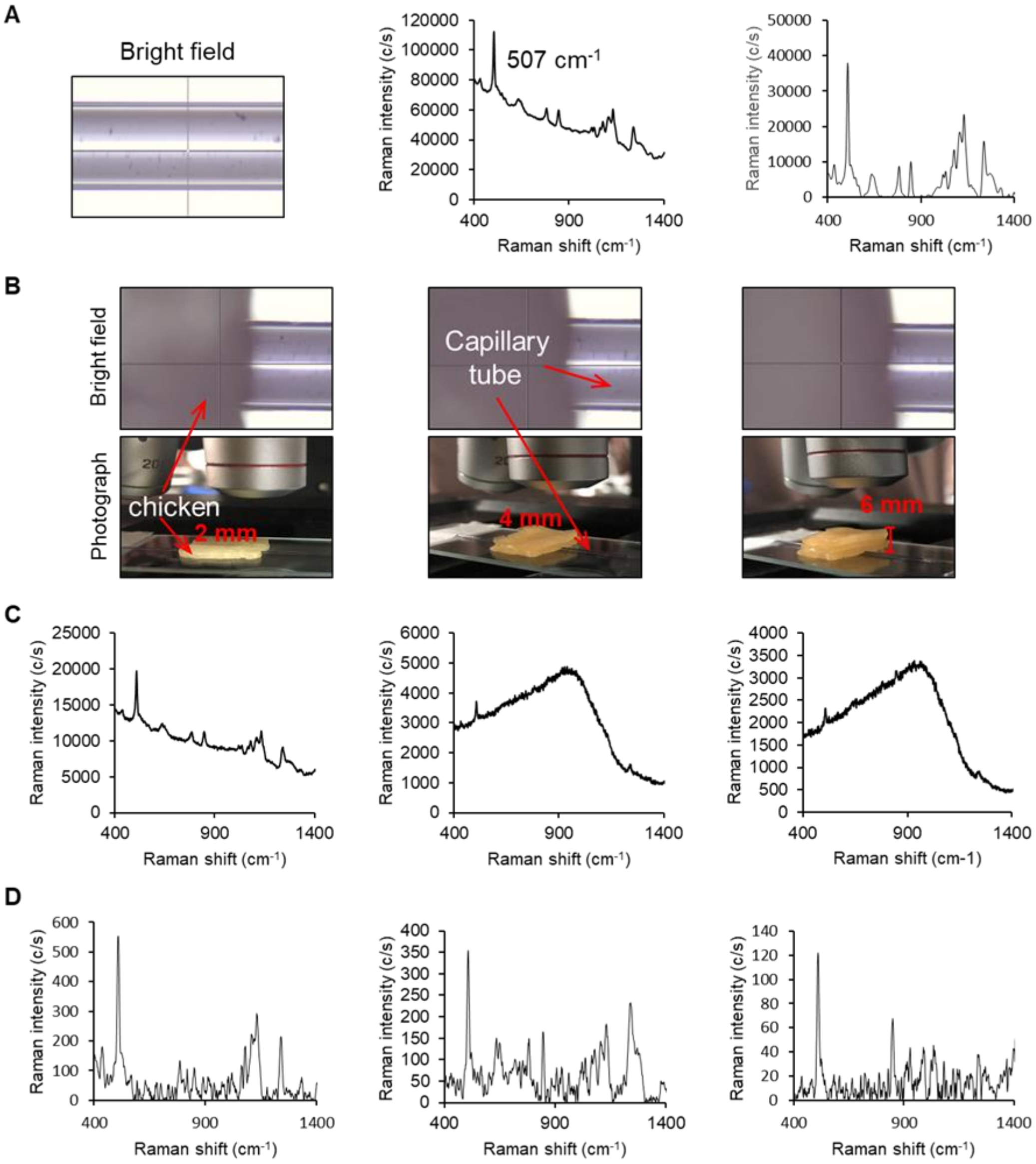

Figure S9. Evaluation of the Raman signal penetration depth. (A) Bright field image of the capillary tube with Au-Ur@DTTC nanoparticles and the corresponding Raman spectrum before and after baseline being subtracted. (B) Bright field images and photographs of the capillary tube with Au-Ur@DTTC nanoparticles covered with different thickness of chicken. (C) The corresponding Raman spectra of Au-Ur@DTTC nanoparticles covered with different thickness (2 mm, 4 mm and 6 mm) of chicken before and after baseline subtracted. The spectra were collected under 785 nm laser with a laser power of 150 mW, 5 × objective, integration time of 0.5 s.

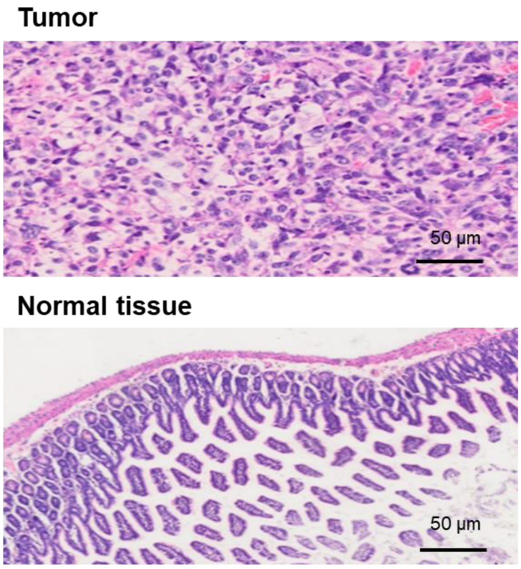

Figure S10. Histology images of the hematoxylin-eosin (H&E) stained orthotopic SKOV3 tumor and its surrounding intestine tissue.

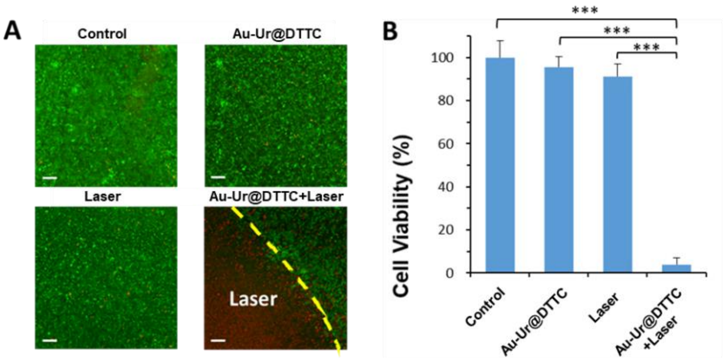

Figure S11. ***In vitro*** **photothermal therapy of SKOV3 ovarian cancer cells using Au-Ur@DTTC NPs.** (A) Calcein AM/ propidium iodide (PI) stained samples show viability of the SKOV3 cells due to laser irradiation (808 nm, 1.50 W/cm^2 for 5 min) with or without administration of the nanoparticles. Laser irradiation was initiated 4 hours after addition of the nanoparticles to the cells. Calcein AM stains live cells (green color), and PI stains dead cells (red color). Scale bars = 50 µm, yellow dotted line denote the boundary of the laser. (B) Quantified cell viability of SKOV3 ovarian cancer cells in response to Au-Ur@DTTC nanoparticles and laser irradiation. (n = 5, ***p < 0.001 relative to control).

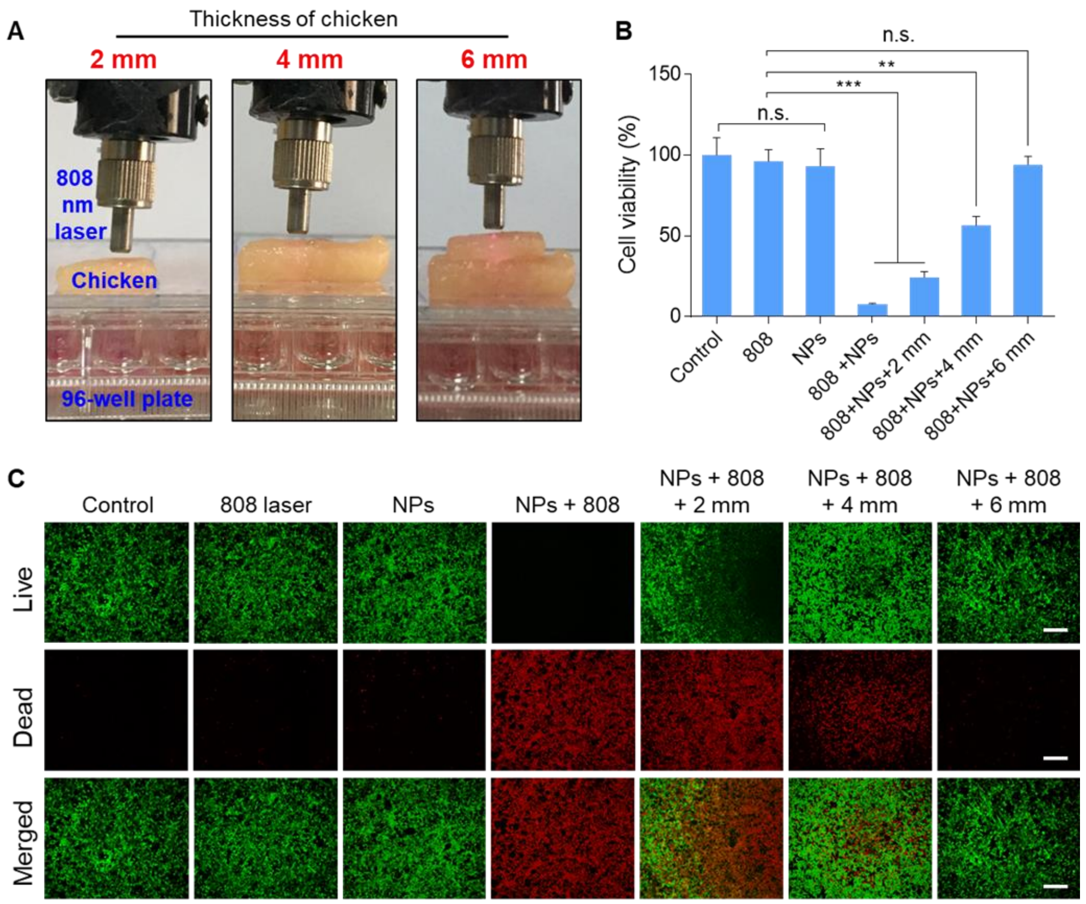

Figure S12. Evaluation of the penetration depth of photothermal therapy. (A) Representative photographs of photothermal therapy effect analysis for SKOV3 tumor cells with different thickness of chicken covered. (B) Quantified cell viability of SKOV3 cells in response to Au-Ur@DTTC nanoparticles (OD 50) and laser irradiation (808 nm 1.5 W cm^{-2}, 5 min) with chicken (0, 2, 4, and 6 mm) overlay. (C) The corresponding calcein AM/ propidium iodide (PI) staining images of SKOV3 cells after various treatments. (Scale bar = 100 μm, n = 5, ***p* < 0.01, ****p* < 0.001)

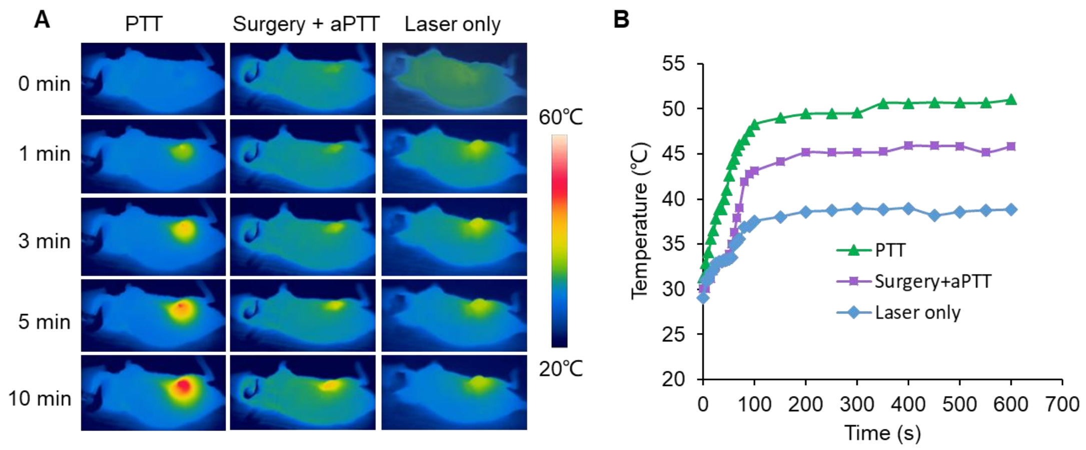

Figure S13. Tumor temperature during PTT, Surgery + aPTT, and laser only treatments. (A) Thermal images of subcutaneous SKOV3 tumor-bearing mice treated with PTT, surgery+aPTT, and laser only during laser irradiation at different time intervals. (1.5 W/cm^2, 10 min). (B) The temperature change curves of tumor site of mice in groups of PTT, surgery+aPTT, and laser only during laser irradiation, (n = 5).

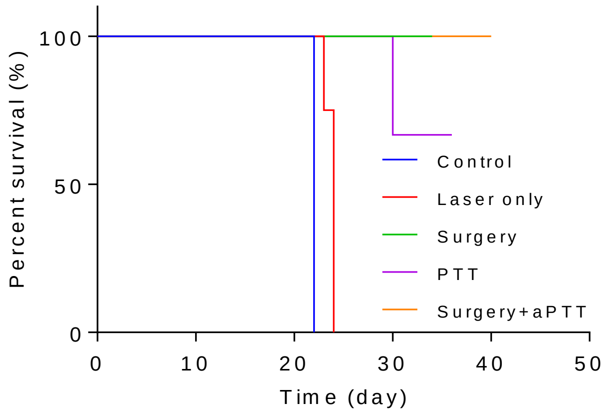

Figure S14. Kaplan–Meier plot of the orthotopic SKOV3 tumor-bearing mice post-treatments. The orthotopic SKOV3 tumor-bearing mice treated with Surgery + aPTT exhibit the highest survival rate. *p* values were calculated by Student t-test (***p* <0.01 relative to control mice).

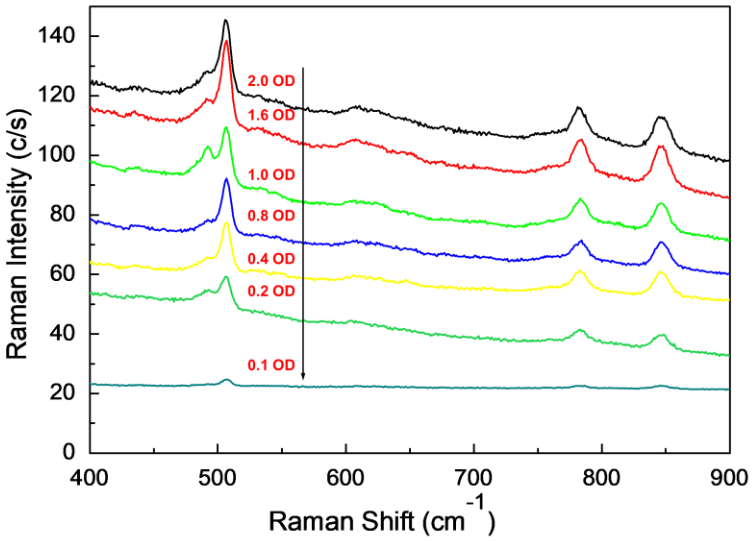

Figure S15. The detection limit of the handheld Raman system. Au-Ur@DTTC NPs solution was diluted to a series of concentrations (0.1~2 OD), then detected the Raman signals of the diluted solutions separately.

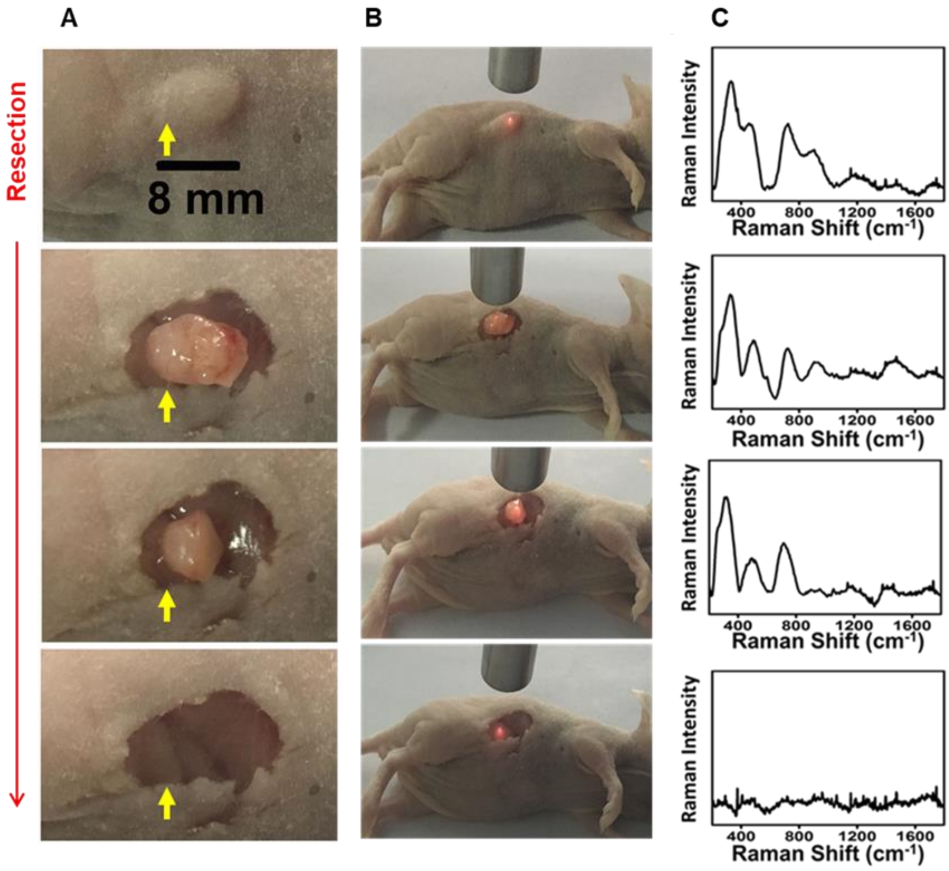

Figure S16. Intraoperative tumor resection using a handheld Raman probe as the guide during the surgery. (A, B) Photographs showing the experimental setup for using the handheld Raman probe to detect the tumor boundaries in a mouse with SKOV3 ovarian tumor xenograft. (C) Characteristic Raman spectra of the nanoparticles embedded within the tumors, at different stages of the SERS-guided tumor resection surgery.

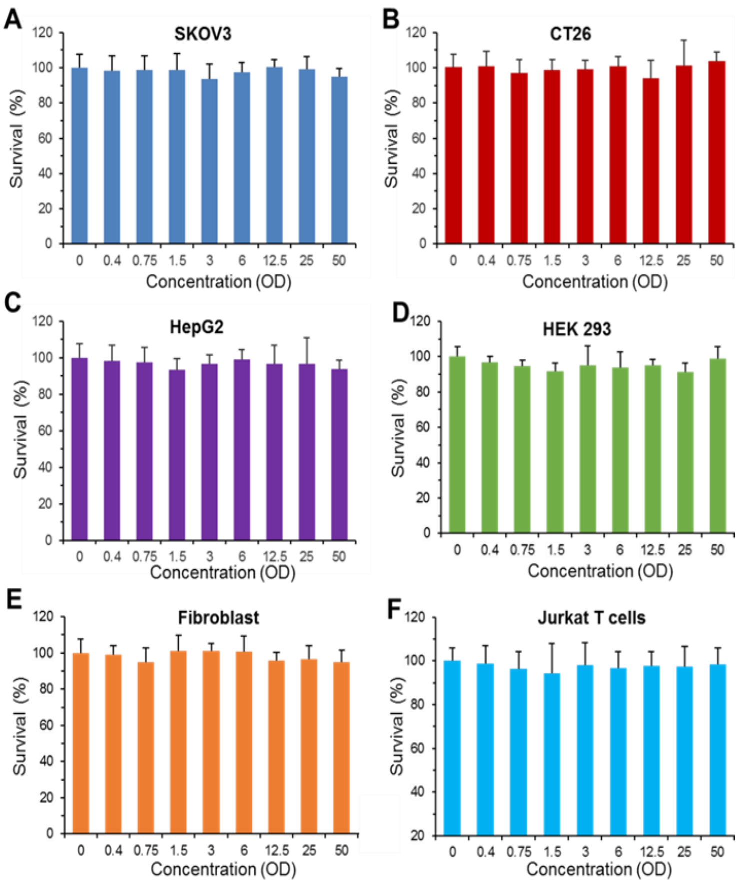

Figure S17. Cytoxicity of Au-Ur@DTTC NPs. (A-F) Viability of SKOV3, CT26, HepG2, HEK 293, Fibroblasts, and Jurkat T cells incubation with different concentrations (0-50 OD, optical density) of Au-Ur@DTTC nanoparticles for 72 h.

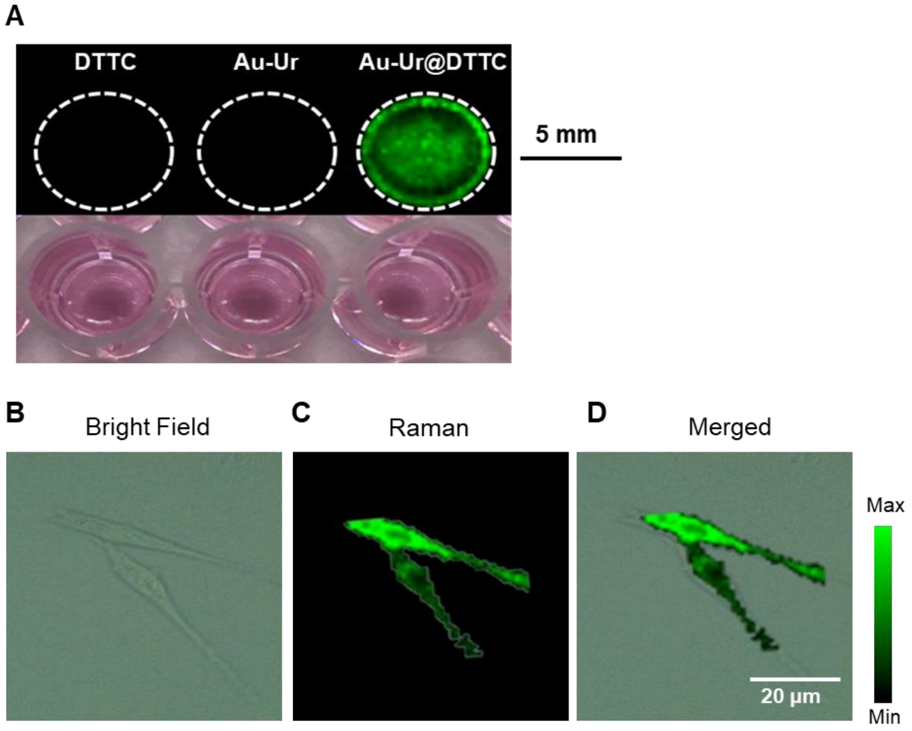

Figure S18. ***In vitro* Raman imaging.** (A) Raman image of the SKOV3 cells incubated with Au-Ur, DTTC, and Au-Ur@DTTC nanoparticles in a 96-well dish (imaging parameters: 785 nm laser, 75 mW, 5× objective, integration time of 0.5 s, and ~10 min in total). (B) Bright field, (C) Raman and (D) merged images of the SKOV3 cells after incubation with Au-Ur@DTTC NPs for 12 h (75 mW, 20× objective, integration time of 0.5 s, and ~15 min in total). Cells were washed by PBS for 3 times before imaging.

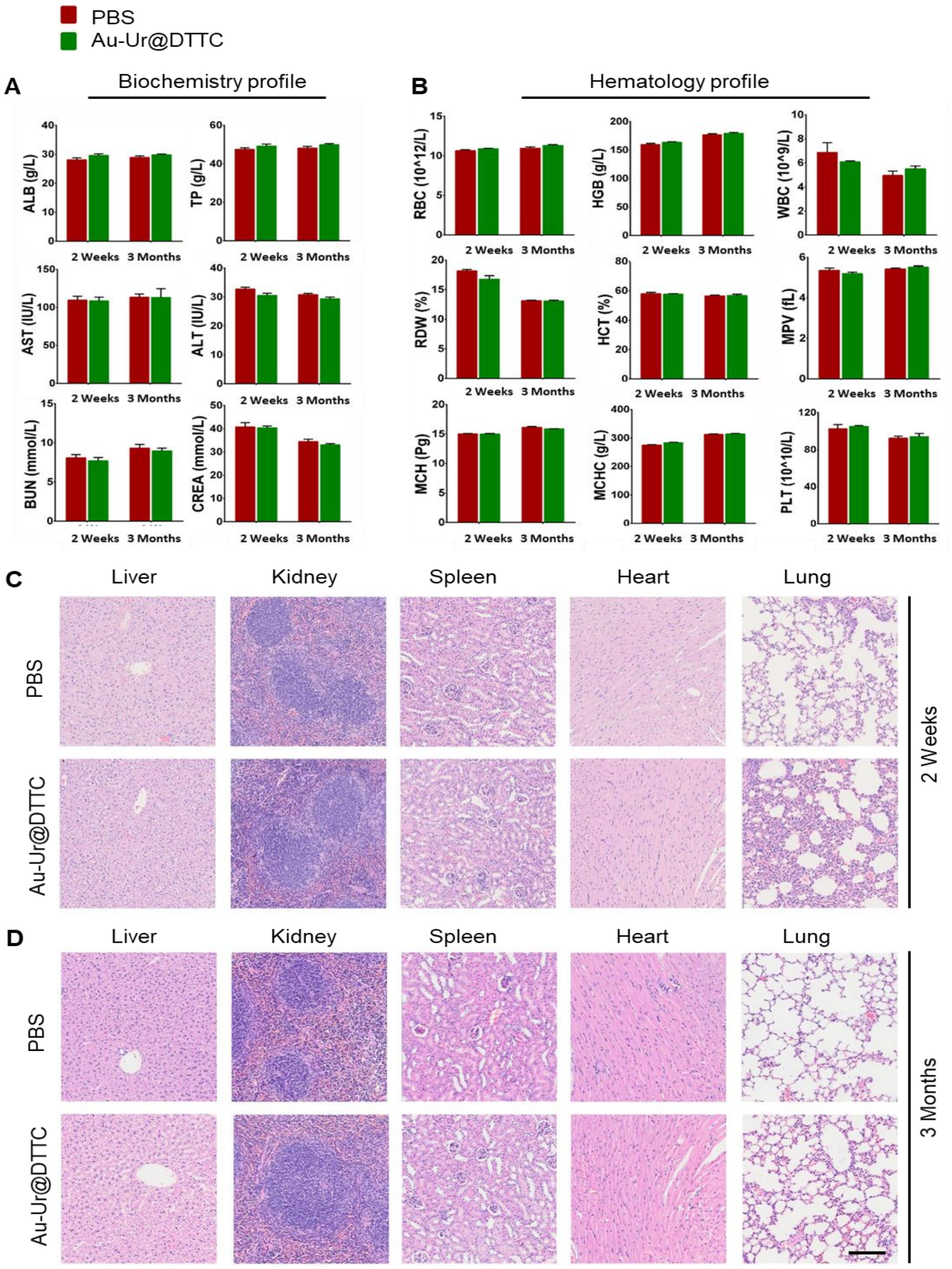

Figure S19. ***In vivo* toxicity evaluation.** (A) Haematological data of the mice intravenously injected with PBS (n = 5) or Au-Ur@DTTC nanoparticles in PBS (n = 5, 0.2 g/Kg) at 2 weeks and 3 months post-injection times. (B) Biochemical analyses of the blood samples obtained 2 weeks and 3 months after injection of the PBS and Au-Ur@DTTC nanoparticles. No statistical significance were found in all analyses. (C, D) Histological images of the hematoxylin and eosin (H & E) stained liver, kidney, spleen, heart, and lung tissue-sections obtained at 2 weeks and 3 months post-injection from mice injected with PBS or Au-Ur@DTTC nanoparticles (scale bars: 100 μm for all panels).
